# Reprograming of proteasomal degradation by branched chain amino acid metabolism

**DOI:** 10.1111/acel.13725

**Published:** 2022-09-27

**Authors:** Sonia Ravanelli, Qiaochu Li, Andrea Annibal, Aleksandra Trifunovic, Adam Antebi, Thorsten Hoppe

**Affiliations:** ^1^ Institute for Genetics University of Cologne Cologne Germany; ^2^ Cologne Excellence Cluster on Cellular Stress Responses in Aging‐Associated Diseases (CECAD) University of Cologne Cologne Germany; ^3^ Max Planck Institute for Biology of Ageing Cologne Germany; ^4^ Center for Molecular Medicine Cologne (CMMC) University of Cologne Cologne Germany; ^5^ Institute for Mitochondrial Diseases and Ageing, Medical Faculty University of Cologne Cologne Germany

**Keywords:** aging, branched‐chain amino acid, branched‐chain aminotransferase, *Caenorhabditis elegans*, metabolism, proteasome, proteostasis, ubiquitin

## Abstract

Branched‐chain amino acid (BCAA) metabolism is a central hub for energy production and regulation of numerous physiological processes. Controversially, both increased and decreased levels of BCAAs are associated with longevity. Using genetics and multi‐omics analyses in *Caenorhabditis elegans*, we identified adaptive regulation of the ubiquitin‐proteasome system (UPS) in response to defective BCAA catabolic reactions after the initial transamination step. Worms with impaired BCAA metabolism show a slower turnover of a GFP‐based proteasome substrate, which is suppressed by loss‐of‐function of the first BCAA catabolic enzyme, the branched‐chain aminotransferase BCAT‐1. The exogenous supply of BCAA‐derived carboxylic acids, which are known to accumulate in the body fluid of patients with BCAA metabolic disorders, is sufficient to regulate the UPS. The link between BCAA intermediates and UPS function presented here sheds light on the unexplained role of BCAAs in the aging process and opens future possibilities for therapeutic interventions.

## Graphical abstract

Using genetics and multi‐omics analyses in *Caenorhabditis elegans*, we identified adaptive regulation of the ubiquitin‐proteasome system (UPS) in response to defective branched chain amino acid (BCAA) metabolism. Worms with impaired BCAA metabolism show slower turnover of a GFP‐based proteasome substrate, which is suppressed by loss‐of‐function of the first BCAA catabolic enzyme, the branched chain aminotransferase BCAT‐1. Exogenous supply of BCAA‐derived carboxylic acids is sufficient to regulate the UPS.

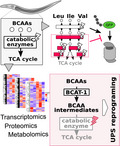

## 1 INTRODUCTION

The branched‐chain amino acids (BCAA) leucine, isoleucine, and valine are essential for the health of the organism and cannot be synthesized by animals. BCAA catabolism is highly conserved, and the first limiting step in the formation of branched‐chain α‐ketoacids is mediated by the branched‐chain aminotransferase BCAT. Complete oxidation of leucine, valine, and isoleucine results in the formation of acetyl‐CoA, succinyl‐CoA, or both (Neinast et al., 2019). In addition to its importance as a catabolic pathway, BCAA metabolism is thought to have numerous other physiological functions, including a prominent role in the regulation of physiological aging (Biswas et al., 2019; Neinast et al., 2019; Trautman et al., 2022). However, the mechanistic role of BCAAs and derivatives in healthy aging remains controversial. BCAAs have been considered for a long time as beneficial supplements, especially in the elderly and athletes to counteract frailty and muscle damage, respectively (Trautman et al., 2022; Valerio et al., 2011). Accordingly, BCAA supplementation has been shown to extend lifespan in male mice (D'Antona et al., 2010) and nematodes (Mansfeld et al., 2015). In contrast, recent studies reported that supplementation of BCAAs reduced lifespan in mice (Solon‐Biet et al., 2019), while BCAA dietary restriction improved longevity both in mice (Richardson et al., 2021; Solon‐Biet et al., 2019) and flies (Juricic et al., 2020), suggesting that BCAA dietary restriction might be a promising intervention strategy to promote healthy aging (Trautman et al., 2022).

Inborn metabolic errors caused by defects in enzymes involved in the biochemical BCAA pathway after initial transamination, such as maple syrup urine disease (MSUD), isovaleric acidemia (IVA), 2‐methylbutyryl‐CoA dehydrogenase deficiency (MBDD), and isobutyryl‐CoA dehydrogenase deficiency (IBDD), are classified as organic aciduria or organic acidemia because of elevated levels of short‐chain carboxylic acids in either urine or blood (Villani et al., 2017). Organic aciduria presents a wide range of symptoms, often associated with neurological impairment (Villani et al., 2017; Wajner et al., 2020). Classic therapeutic interventions consist of dietary restriction to reduce BCAA metabolic flux and supplementation with compounds such as carnitine that promote the excretion of toxic metabolic byproducts (Villani et al., 2017). Although the morbidity and mortality of organic aciduria are significantly reduced by current treatments, long‐term systemic and neurologic damage are rarely prevented (Wajner et al., 2020).

Altered BCAA metabolism has also been linked to neurodegenerative diseases, diabetes, and cancer (Biswas et al., 2019; Conway, 2020; Neinast et al., 2019; Peng et al., 2020; White & Newgard, 2019), all of which are known to be characterized by changes in protein homeostasis (proteostasis) (Labbadia & Morimoto, 2015; Neinast et al., 2019; Ottens et al., 2021). The ubiquitin‐proteasome system (UPS) plays a central role in maintaining proteostasis by degrading damaged, superfluous, and potentially toxic proteins (Pohl & Dikic, 2019). After ubiquitylation, catalyzed by an enzymatic cascade of ubiquitin‐activating enzymes (E1), ubiquitin‐conjugating enzymes (E2), and ubiquitin ligases (E3), UPS substrates are directed for degradation to the 26S proteasome, which consists of a central proteolytic core complex (20S) and two regulatory particles (19S) (Bard et al., 2018; Pohl & Dikic, 2019). UPS activity is adapted to cellular needs by modulating proteasome quantity and composition (Rousseau & Bertolotti, 2018). Accordingly, reversible proteasome disassembly is triggered by oxidative stress (Grune et al., 2011; Livnat‐Levanon et al., 2014; Wang et al., 2010) or metabolic disturbances (Meul et al., 2020). Although metabolic defects and proteostasis decline are linked to numerous pathologies and known hallmarks of aging (Lopez‐Otin et al., 2013), the two topics have been mainly examined independently (Ottens et al., 2021).

In light of the conflicting findings regarding the dietary modulation of BCAA levels on longevity, we decided to further explore the complex physiological role of BCAAs with particular attention to proteostasis. In a previous study, we demonstrated that depletion of *ivd‐1*, a mitochondrial enzyme involved in the catabolism of leucine, leads to reduced protein turnover by the UPS, both in *C. elegans* and in mammalian cells (Segref et al., 2014). We decided to inhibit the other catabolic enzymes responsible for the oxidation of BCAAs and monitored the proteolytic activity of the UPS in worms. Instead of modulating the BCAA intake, we aimed to study the UPS regulation upon altered BCAA metabolic flux. Combining genetics with multi‐omics analyses, we observed that only defects in the BCAA catabolism occurring downstream of initial transamination reduced the turnover rate of a GFP‐based proteasome substrate, which was fully restored in *bcat‐1* loss‐of‐function mutants. Our data suggest that, only when BCAT‐1 is functional, does impaired downstream BCAA metabolic flux trigger an adaptive transcriptional response involving downregulation of UPS in favor of other proteolytic pathways. Growth medium supplementation experiments showed that BCAA‐derived carboxylic acids known to accumulate in BCAA‐specific organic aciduria were sufficient to reduce ubiquitin‐dependent proteolysis and enhance aggregation of metastable proteins, suggesting a possible role as stress molecules. The causal relationship demonstrated here between BCAA metabolism and reprogramming of proteasomal degradation opens future possibilities for therapeutic interventions in mitochondrial and neurological pathologies, thereby promoting healthy aging.

## 2 EXPERIMENTAL PROCEDURES

### 2.1 *C. elegans* strains and maintenance

*C. elegans* strains were cultured according to standard methods (Stiernagle, 2006), at 20°C, on nematode growth medium (NGM) agar plates seeded with OP50 *E. coli* as a food source. All strains derive from the Bristol N2 strain. The strains *ivd‐1(tm6784) IV* and *hecd‐1 (tm2371) IV* were obtained by National BioResource Project (NBRP); MIR23: *risIs3 [K02A4.1p::K02A4.1::GFP + unc‐119(+)*], FX30253: *tmC24 [F23D12.4(tmIs1233); unc‐9(tm9718)] X; tmEx4950 [unc‐9(+) + vha‐6p::GFP]*, and GF78: *dgEx78 [(pAMS68) vha‐6p::Q40::YFP + rol‐6(su1006) + pBluescript II]* were obtained by Caenorhabditis Genetics Center (CGC); PP563: *unc‐119(ed4); hhIs64 [unc‐119(+); sur‐5p::UbV‐GFP]III* was generated in our lab (Segref et al., 2011). *bcat‐1 (hh58) X* and *bcat‐1 (hh56)/tmC24 [F23D12.4(tmIs1233) unc‐9(tm9718)] X* were generated in this study. To reduce effects caused by genetic backgrounds, all strains were outcrossed at least twice and whenever possible all mutant strains, including wild‐type, analyzed in a single experiment were isolated from the same cross. Genotyping primers are reported in Table S1.

### 2.2 Synchronization of *C. elegans* populations

Unless otherwise stated, all experimental analyses were performed on Day 1 adult worms, synchronized by egg‐prep (Stiernagle, 2006). In brief, worms were washed off the plates with M9, bleached with sodium hypochlorite solution, and, after 3 washing steps with M9, the obtained eggs were seeded on a culture plate. Estimation of the number of eggs was done in triplicates with a 1 μl drop of suspension in M9 to seed appropriate amounts of worms in each plate, considering lethality and fertility defects of each mutant strain. In case of developmental delay, either synchronization or collection/imaging was adapted according to the quantified average generation time of each mutant strain and by optical inspection to confirm that worms reached the egg‐bearing adult stage.

### 2.3 Western blot

Protein lysates were obtained by sonication, boiling at 95°C for 5 min, and centrifugation at 18000*g* for 15 min at 4°C. The supernatant containing either 5 μg of protein (quantified by Pierce BCA protein assay, ThermoFisher Scientific) or the equivalent volume of 20 picked worms was supplemented with an equal amount of 2x SDS loading buffer before loading on Bis‐Tris 4%–12% polyacrylamide gels for electrophoresis. Proteins were transferred to Amersham Protran 0.1 NC nitrocellulose membranes (Cytiva) with a semi‐dry blotting system (Bio‐Rad, Trans‐Blot Turbo) using NuPAGE transfer buffer (Thermo Fischer Scientific). Membranes were blocked with 3% milk (in PBS + 0.1% Tween 20) for at least 20 min and incubated with the primary antibodies overnight at 4°C in RotiBlock (Carl Roth). Membranes were immunoblotted in one step with mouse anti‐GFP and rabbit anti‐tubulin primary antibodies (1 hour at room temperature or overnight at 4°C) and subsequently with Li‐Cor 680RD donkey anti‐rabbit and 800CW donkey anti‐mouse secondary antibodies (1 hour at room temperature). Antibody detection was conducted with a Li‐Cor Odyssey scanner and Image Studio Lite (version 5.2.5) was used to acquire images and quantify band intensities, setting as background the median of the measured intensity on the adjacent area of both sides for each band. All intensities were normalized by dividing for the respective tubulin intensity as a loading control. All quantifications are reported as log_2_ of the normalized intensities divided by the normalized intensity of the control sample as indicated for each figure. Uncropped Western blots and details about used antibodies are reported in Figure S6 and Table S2, respectively.

### 2.4 Gene suppression by RNAi

RNA interference (RNAi) was performed following the standard feeding method, making use of the bacterial *C. elegans* RNAi Collections Ahringer (RRID:SCR_017064) and ORFeomeWS112 (Laboratory of Marc Vidal). Bacteria were grown in LB medium supplemented with 0.1 mg/ml ampicillin overnight, diluted to an optical density (OD)_600_ of 0.1 the following day, and grown to a maximum OD_600_ of 0.9. dsRNA expression was induced by adding IPTG to a final concentration of 2 mM for 30–60 min shaking at 37°C. Bacteria were seeded onto growth media NGM plates containing 2 mM IPTG and 0.1 mg/ml ampicillin. Synchronized eggs were homogenously transferred on the seeded RNAi plates and incubated at 20°C until the worm reached the adult stage for live imaging and/or collection. As a control, bacteria expressing the empty vector pPD129.36 were used. *Ivd‐1* or *hecd‐1*, which are known to accumulate the UbV‐GFP (Segref et al., 2014), served as a positive control for RNAi activation. Alternatively, RNAi efficacy was validated with qRT‐PCR.

### 2.5 Supplementation of BCAA‐derived compounds

25x stock solutions of each metabolite were prepared in H_2_O and poured on NGM plates seeded with OP50 to reach a final concentration of 3, 6, or 12 mM; H_2_O served as control. The growth medium pH was assessed to be above 6 by submerging a pH test strip into the agar. After complete absorption at room temperature, eggs obtained by egg‐prep were seeded on top of the plates and incubated at 20°C until adulthood. Details about each compound are reported in Table S3.

### 2.6 Live microscopy

For visualization of UbV‐GFP, 10–15 worms were picked and mounted on 3% agar pads, immobilizing them with 90 mM sodium azide. Fluorescent and brightfield images of mounted immobilized worms were captured with Zeiss Axiozoom V16, equipped with Axiocam 506mono and ZEN 2.3 software. Fluorescent exposure times were always kept constant for every group of images compared. For protein aggregate quantification, 1‐ or 2‐day‐old adults were placed on plates with or without isovaleric acid supplementation to lay eggs for 4 hours, and transgenic L4 animals were transferred to new plates. Animals were then transferred every 2 days to new plates until aggregates were quantified on day six of adulthood as reported previously (Mohri‐Shiomi & Garsin, 2008).

### 2.7 CRISPR‐Cas9 mutation

Mutation of *bcat‐1* was conducted through CRISPR‐Cas9, following the *dpy‐10* co‐conversion strategy described previously (Paix et al., 2017). Two different guide RNAs (crRNA) were designed to introduce a restriction site for NheI that contains TAG, serving as a premature stop codon in the first exon of the coding sequence, in addition to a frameshift. The detailed procedure is described in the Supplementary Material. Oligonucleotides used for genotyping and CRISPR‐Cas9 editing are reported in Tables S1 and S4, respectively.

### 2.8 Omics analyses

Synchronized worms by egg‐prep were harvested on Day 1 of adulthood after washing with M9 at least twice to remove bacteria. Worm pellets were split into 3 Eppendorf tubes (one for each omics) and flash‐frozen with liquid nitrogen before storing at −80°C. Four replicates were collected in independent experiments, and the following processing was conducted in parallel for each omics analysis. For details about sample and data processing, see the Supplementary Methods.

The RNA raw data, FPKM values, and experimental information have been deposited in NCBI's Gene Expression Omnibus with dataset identifier GSE185451. The mass spectrometry proteomics data have been deposited to the ProteomeXchange Consortium via the PRIDE partner repository with the dataset identifier PXD028286. Metabolomics raw and processed data are reported in Table S5.

### 2.9 Data visualization and statistical analysis

All graphs generated with RStudio (version 1.2.5033, R version 4.0.0) report means and standard deviations and significant difference is displayed as * for *p*‐value ≤0.05 and ** for *p*‐value ≤0.01, calculated with pairwise *t*‐tests or Tukey's Honest Significant Difference (HSD) combined with one‐way ANOVA for normally distributed data. For not normally distributed data, pairwise comparisons were computed using the Wilcoxon rank sum test with Bonferroni adjustment of *p*‐values for multi‐testing.

## 3 RESULTS

### 3.1 Impaired BCAA transamination and downstream catabolic reactions affect the UPS in opposite ways

**FIGURE 1:**
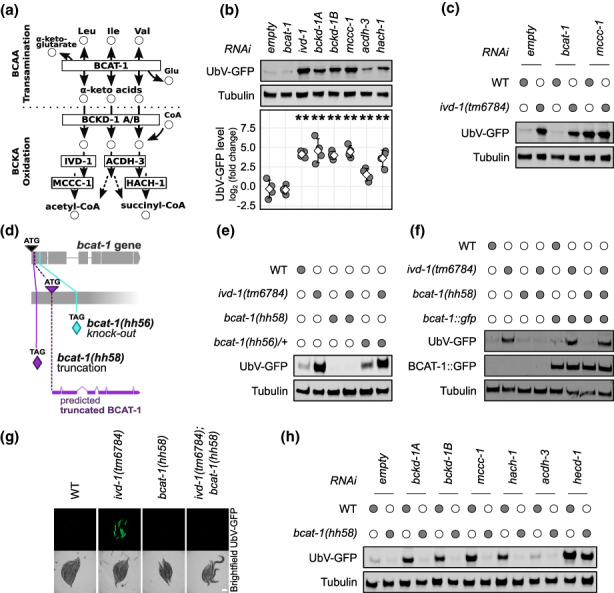
Impaired BCAA transamination and downstream catabolic reactions affect the UPS in opposite ways. (a) BCAA metabolism schematic view with metabolites as circles and enzymes as boxes. (b, c, e, f, h) Western blot analysis of adult worm lysates immunoblotted for GFP (UbV‐GFP and BCAT‐1::GFP) and tubulin as a loading control. (b) Worms were treated with RNAi against indicated genes, empty RNAi vector (empty) as control. Micrographs in Figure S1a. Below the blot, quantification of four experimental replicates displayed as the log_2_ of the UbV‐GFP intensity normalized on tubulin and relative to the wild‐type (WT) control. Means as white squares and standard deviations as error bars. A statistically significant difference in relation to control was calculated with pairwise t‐tests and indicated with ** for *p*‐value ≤0.01. (c) Wild‐type (WT) and *ivd‐1(tm6784)* worms, indicated with filled circles, were treated with RNAi against *bcat‐1*, *mccc‐1*, or empty RNAi vector (empty) as control. Micrographs and *bcat‐1* transcript quantification in Figure S1a,b. Three experimental replicates are quantified in Figure S1c. (d) Overview of the CRISPR‐Cas9 *bcat‐1* mutations, with indicated premature stop codons (TAG) for the *hh56* allele (light blue) and *hh58* allele (violet). (e) Filled circles denote genetic mutations, *bcat‐1(hh56)/+* indicates heterozygous mutation balanced with *tmC24*. Micrographs in Figure S1f. Three experimental replicates quantified in Figure S1g. (f) Filled circles denote genetic mutations or *bcat‐1::gfp* transgene expression. Three experimental replicates are quantified in Figure S2b. (g) Fluorescent and brightfield micrographs of immobilized worms, relative to (f), scale bar: 200 μm. Micrographs of *bcat‐1::gfp* rescue lines in Figure S2a. (h) WT and *bcat‐1(hh58)* worms, indicated with filled circles, were treated with RNAi against indicated genes, empty RNAi vector (empty) as control. Micrographs in Figure S2f. Four experimental replicates are quantified in Figure S2g.

Considering that impaired BCAA metabolism is associated with numerous pathological conditions characterized by altered proteostasis (Biswas et al., 2019; Conway, 2020; Neinast et al., 2019; Ottens et al., 2021; White & Newgard, 2019), we wondered whether enzymatic regulation of the BCAA biochemical pathway (Figure 1a) directly affects UPS activity. Therefore, we suppressed enzymes required for BCAA catabolism in *C. elegans* by RNAi and monitored the turnover of a GFP‐based ubiquitin fusion degradation (UFD) substrate, termed UbV‐GFP, which is a simple, but powerful method for monitoring UPS functionality in vivo (Segref et al., 2011). Indeed, suppression of several mitochondrial enzymes responsible for the oxidative degradation of BCAAs, namely *ivd‐1, bckd‐1A, bckd‐1B*, *mccc‐1*, *acdh‐3*, and *hach‐1*, resulted in the accumulation of UbV‐GFP, suggesting reduced UPS function (Figure 1b; Figure S1a). Surprisingly, inhibition of the first enzymatic step of BCAA metabolism by *bcat‐1(RNAi)* did not affect substrate turnover (Figure 1b; Figure S1a). These results suggest that disruption of BCAA transamination and downstream catabolic steps induce opposite effects on the turnover of proteasome substrates.

To constitutively affect BCAA catabolism downstream of BCAT‐1, we used the loss‐of‐function mutant *ivd‐1(tm6784)*, which showed a strong accumulation of UbV‐GFP (Figure 1c), similarly to its RNAi suppression and of the other BCAA catabolic enzymes tested (Figure 1b; Figure S1a). Despite being upstream in the BCAA pathway (Figure 1a), *bcat‐1(RNAi)* did not abrogate the UbV‐GFP accumulation of *ivd‐1(tm6784)* (Figure 1c; Figure S1b,c). However, the low amount of *bcat‐1* transcript detected by qRT‐PCR suggests that RNAi treatment does not completely downregulate BCAT‐1 activity (Figure S1b). Therefore, we genetically modified the endogenous *bcat‐1* locus using CRISPR‐Cas9 technology (Paix et al., 2017). To completely knock down the function of BCAT‐1, we engineered alternative guide RNAs that introduce a premature stop codon into the first exon of the *bcat‐1* genomic locus and obtained two distinct *bcat‐1* alleles (Figure 1d). Previous data on *bcat‐1* RNAi depletion revealed partial embryonic lethality (Rual et al., 2004). Accordingly, the allele with the more distant premature stop codon, *hh56*, was 100% embryonically lethal (Figure S1d), indicating a complete loss of function of *bcat‐1*. In contrast, the allele *hh58* was homozygously viable, likely due to an alternative start codon downstream of the premature stop codon (Figure 1d). In support of this hypothesis, the transcript level of *bcat‐1* was not reduced in the *bcat‐1(hh58)* mutants (Figure S1e).

Like *bcat‐1(RNAi)*, neither *bcat‐1* mutant allele induced accumulation of the UbV‐GFP substrate protein (Figure 1e; Figure S1f,g). However, *bcat‐1(hh58)* was able to suppress the substrate degradation defect of *ivd‐1(tm6784)*, which was confirmed by transgenic rescue with *bcat‐1::gfp* expression (Mansfeld et al., 2015) (Figure 1f,g; Figure S2a,b). Although viable, the *bcat‐1(hh58)* allele exhibited distinct physiological defects: *bcat‐1(hh58)* mutant worms produced roughly 80% fewer viable offspring compared with wild‐type (Figure S2c), took approximately 1 day longer to reach adulthood (Figure S2d), and only 50% of their laid eggs eventually hatched (Figure S2e). All of these impairments were completely rescued by expression of *bcat‐1::gfp* (Mansfeld et al., 2015), suggesting that *bcat‐1(hh58)* mutation causes a loss of function of *bcat‐1*. Of note, *bcat‐1(hh58)* was able to suppress UbV‐GFP accumulation induced by RNAi suppression of downstream enzymes, whereas UPS defects caused by depletion of the E3 ligase *hecd‐1*, which is known to ubiquitylate the UbV‐GFP substrate (Segref et al., 2014), were only slightly reduced (Figure 1h; Figure S2f,g). These results suggest that impairments in BCAA metabolism downstream of the first transamination step negatively affect ubiquitin‐dependent degradation; however, inhibition of the BCAA catabolic pathway by BCAT‐1 loss‐of‐function may reverse this effect.

### 3.2 Altered BCAA metabolism activates an adaptive transcriptional stress response

**FIGURE 2:**
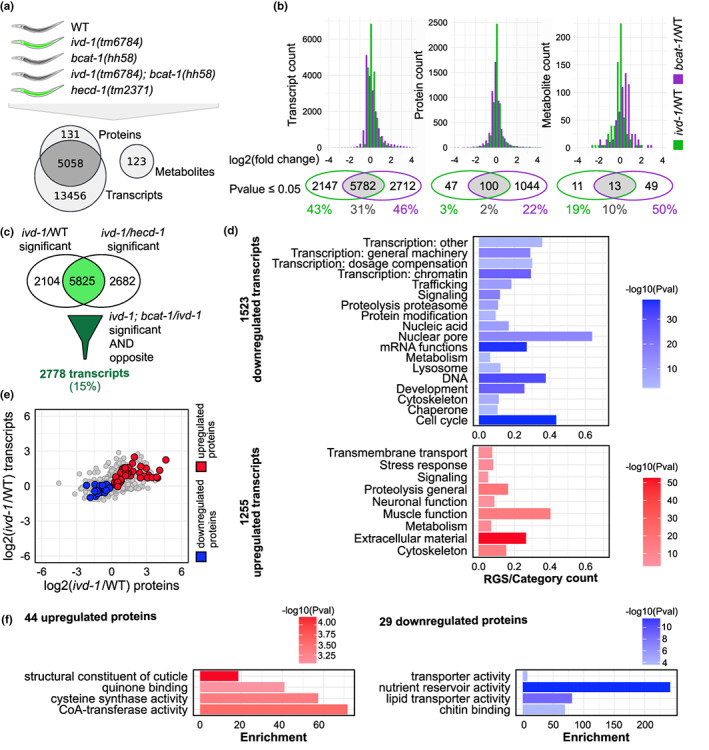
Altered BCAA metabolism activates an adaptive transcriptional stress response. (a) Mutant strains included in the multi‐omics analysis and their effect on UbV‐GFP turnover (top), Venn diagram of detected transcripts, proteins, and metabolites (bottom). Four experimental replicates. Heatmaps of the complete datasets in Figure S3. (b) Histograms of log_2_(*ivd‐1(tm6784)*/wild‐type) (green) and log_2_(*bcat‐1(hh58)*/wild‐type) (violet), with respective Venn diagrams of significantly regulated transcripts, proteins, and metabolites (*p*‐value ≤0.05), 0.3 bin width. Percentages for each dataset are indicated. (c) *ivd‐1(tm6784)‐*significantly regulated transcripts compared to wild‐type (WT) and *hecd‐1(tm2371)* (light green), which are suppressed by *bcat‐1(hh58)* (dark green) (*p*‐value ≤0.05). Relative scatterplot in Figure S4c. (d) Wormcat gene enrichment analysis of downregulated (blue) and upregulated (red) transcripts identified in (c), reported as the proportion of regulated gene set (RGS) for each category, and relative –log_10_ (*p*‐value) (color scale). (e) Scatterplot of log_2_(*ivd‐1/*wild‐type) of proteins and their respective transcripts, with significantly upregulated proteins in red and downregulated in blue which are reversed by *bcat‐1(hh58)* (*p*‐value ≤0.05), as in Figure S4g. (f) GOrilla gene ontology enrichment analysis relative to identified regulated proteins in (e), reported as enrichment score and –log_10_ (*p*‐value) (color scale).

Given the unexpected opposing effects of impaired BCAA transamination and downstream catabolic steps in UPS regulation, we investigated the underlying metabolic regulation. We performed a multi‐omics analysis and compared transcriptome, proteome, and metabolome data from *ivd‐1(tm6784)* and *bcat‐1(hh58)* single and double mutants (Figure 2a; Figure S3). To specifically investigate the role of BCAA metabolism in proteostasis and particularly in the regulation of the UPS, we included the E3 ligase mutant *hecd‐1(tm2371)*, which completely blocks poly‐ubiquitylation of the UbV‐GFP substrate (Segref et al., 2014). In contrast with other UPS components, depletion of the E3 ligase HECD‐1 as positive control is particularly suitable to specifically block the ubiquitylation of proteasome substrates that resemble the UbV‐GFP and avoid proteostasis collapse due to broader UPS impairment. Although both *ivd‐1(tm6784)* and *hecd‐1(tm2371)* mutants caused UbV‐GFP accumulation, their transcriptome and proteome profiles differed greatly (Figure S3), suggesting that the cause of UPS impairment and the associated compensatory response programs are different. Quantification of fold changes revealed stronger reprogramming associated with the *bcat‐1(hh58)* mutation compared with *ivd‐1(tm6784)* at both transcript, protein, and metabolite levels, reflecting the physiological importance of BCAA transamination (Figure 2b; Figure S1d and S2c–e).

To identify the molecular mechanisms underlying the opposite roles of BCAT‐1 and IVD‐1 in UbV‐GFP substrate turnover, we focused on changes caused by *ivd‐1(tm6784)* that were suppressed by *bcat‐1(hh58)*. Despite a general correlation between the regulation mediated by *ivd‐1(tm6784)* and *bcat‐1(hh58)* (Figure S4a), numerous changes associated with the *ivd‐1(tm6784)* mutants were reversed in the *ivd‐1(tm6784); bcat‐1(hh58)* double mutants (Figure S4b). This trend suggests that the restored UPS activity in *ivd‐1(tm6784)*; *bcat‐1(hh58)* is mediated by an adaptive response rather than broad *bcat‐1(hh58)*‐dependent regulation. To identify regulatory pathways that are causally linked and not a consequence of *ivd‐1(tm6784)* proteolytic defects, we selected only *ivd‐1(tm6784)* transcriptional changes compared to both wild‐type and *hecd‐1(tm2371)* (Figure 2c; Figure S4c). Gene set enrichment analysis of the identified transcripts revealed, on one hand, downregulation of genes involved in transcription processes, development, cell cycle, and on the other hand, upregulation of genes involved in stress response, signal transduction, muscles, neurons, and extracellular material (Figure 2d). Proteostasis‐related pathways were also significantly enriched; in particular, the “proteolysis proteasome” category was downregulated in *ivd‐1(tm6784)*, reflecting well the UbV‐GFP substrate turnover (Figure 1f, 2d). In contrast, “proteolysis general” was upregulated (Figure 2d), including several classes of peptidases, such as metallopeptidases and carboxypeptidases (Figure S4d), suggesting a compensatory stress response to impaired BCAA catabolism. Interestingly, six 19S proteasome subunits were downregulated in *ivd‐1(tm6784)* and restored in *bcat‐1(hh58)*, suggesting reprogramming of substrate‐specific recognition rather than general proteasome repression (Figure S4d). Indeed, the two 19S regulatory particles mediate the recognition and translocation of ubiquitylated proteins to the 20S core (Bard et al., 2018). In particular, the RPN‐6.1 subunit is required for proteasomal function in proteostasis and longevity (Vilchez et al., 2012). This suggests a functional link between BCAA catabolism and proteasomal activity.

We also considered the independent contribution of *bcat‐1(hh58)* by selecting transcript changes caused by the *bcat‐1(hh58)* mutation in either the wild‐type or *ivd‐1(tm6784)* background following the same type of regulation (Figure S4e). Few of the identified *ivd‐1(tm6784)*‐regulated transcripts were also found for the *bcat‐1(hh58)*‐specific response (Figure S4f), supporting the idea that the restored UPS activity in *ivd‐1(tm6784)*; *bcat‐1(hh58)* double mutants is not caused by the *bcat‐1(hh58)* mutation alone. Almost all regulated genes that were categorized as components of the UPS were downregulated in *ivd‐1(tm6784)* and specifically upregulated in *ivd‐1(tm6784); bcat‐1(hh58)* double mutants, whereas *bcat‐1(hh58)* mutants were more similar to wild‐type (Figure S4d). These results suggest that impaired BCAA catabolism downstream of the first transamination step triggers a stress response that transcriptionally downregulates UPS components and favors other proteolytic pathways.

### 3.3 BCAA metabolism influences protein degradation and central metabolic pathways

The regulatory effect of *ivd‐1(tm6784)* on the proteome was much weaker than on the transcriptome, with only 3% significant changes compared with 43% (Figure 2b). Therefore, we hypothesize that impaired BCAA metabolism downstream of the transamination step induces a specialized proteolytic adaptation to regulate the level of specific protein classes. To investigate what type of proteins might be differently degraded, we selected proteins that were upregulated or downregulated in *ivd‐1(tm6784)* and reversed by *bcat‐1(hh58)* and compared their levels with the corresponding transcripts (Figure 2e). We also included proteins regulated by *hecd‐1(tm2371)* to detect possible common proteasome substrates (Figure S4g). Although several of the identified proteins were also regulated at the transcript level, more extensive regulation at the protein level was evident (Figure 2e). Gene ontology (GO) enrichment analysis showed that upregulated proteins, possibly protected from degradation, included cuticle components, quinone binding partners, enzymes with CoA‐transferase activity, and cysteine synthases (Figure 2f). On the contrary, proteins involved in nutrient storage, lipid transport, and binding partners of chitin were downregulated and possibly degraded more efficiently. The high number of proteins specifically regulated by *bcat‐1(hh58)* suggests that the proteome is strongly affected by the loss of BCAA transamination (Figure S4h). However, few of the proteins regulated by *bcat‐1(hh58)* were also regulated by *ivd‐1(tm6784)* and repressed by *bcat‐1(hh58)* (Figure S4i), indicating that the adaptive response activated in the presence of impaired BCAA catabolism downstream of initial transamination is distinct from the general physiological effects caused by the *bcat‐1(hh58)* mutation.

**FIGURE 3:**
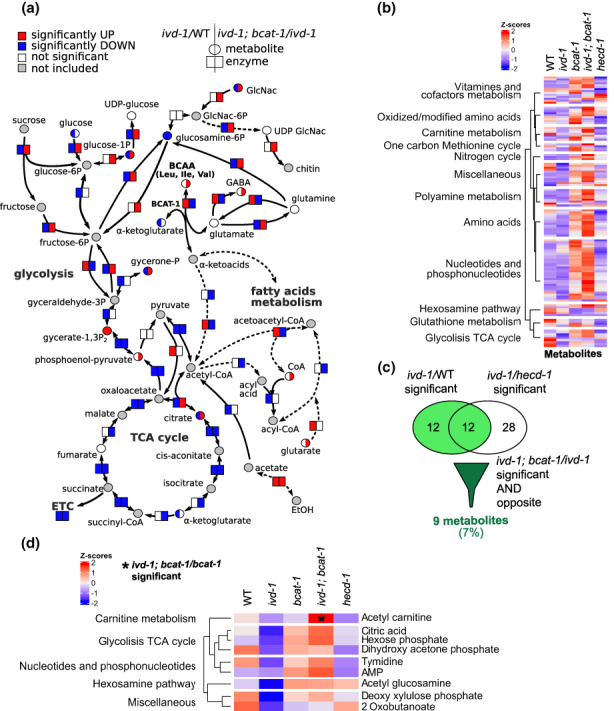
BCAA metabolism influences protein degradation and central metabolic pathways. (a) Metabolic map generated and modified from Pathview with log_2_(*ivd‐1(tm6784)/*wild‐type) (left) and log_2_(*ivd‐1(tm6784)*; *bcat‐1(hh58)/ivd‐1(tm6784))* (right) of metabolites (circles) and enzyme transcripts (rectangles). Colors indicate significant increase (red, *p*‐value ≤0.05), decrease (blue, *p*‐value ≤0.05), not significant change (white, *p*‐value >0.05), or not present in the dataset (gray). (b) Heatmap with Z‐scores of all detected metabolites split into categories. The reported mutant alleles are *ivd‐1(tm6784), bcat‐1(hh58)*, and *hecd‐1(tm2371)*. (c) Venn diagram of *ivd‐1(tm6784)‐*significantly regulated metabolites compared to wild‐type (WT) in light green and *hecd‐1(tm2371)*, which are suppressed by *bcat‐1(hh58)* (dark green) (*p*‐value ≤0.05). (d) Heatmap with Z‐scores of regulated metabolites identified in (c). * highlights significant difference in *ivd‐1(tm6784); bcat‐1(hh58)* compared with *bcat‐1(hh58)* (*p*‐value ≤0.05).

Consistent with its crucial physiological role, BCAA metabolism affected several central metabolic pathways (Figure 3a). In particular, *ivd‐1(tm6784)* exhibited low glycolysis and low TCA cycle. While glycolysis was restored by *bcat‐1(hh58)*, the TCA cycle was further reduced in the *ivd‐1(tm6784); bcat‐1(hh58)* double mutants. Transcription of genes involved in mitochondrial metabolism was unchanged in *ivd‐1(tm6784)*, whereas it was greatly reduced in the *bcat‐1(hh58)* single and *ivd‐1(tm6784); bcat‐1(hh58)* double mutants, which was mainly reflected at the protein level (Figure S5a). Genes involved in other metabolic pathways such as lipid, nucleotide, and amino acid metabolism were both up‐ and downregulated by *ivd‐1(tm6784)* and *bcat‐1(hh58)*, reflecting a more complex metabolic reprogramming. At the metabolite level, the *bcat‐1(hh58)* mutation upregulated numerous metabolites, whereas the *ivd‐1(tm6784)* metabolic profile was more similar to that of the wild‐type control (Figure 3b). We conclude that defective transamination of BCAAs triggers profound metabolic reprogramming, whereas downstream catabolic defects have milder effects. Accordingly, 46 metabolites were specifically regulated by *bcat‐1(hh58)* (Figure S5b,c), whereas only 9 metabolites were regulated by *ivd‐1(tm6784)* and reversed by *bcat‐1(hh58)* (Figure 3c,d). Among these metabolites, acetyl‐carnitine was the only metabolite that was significantly different in *ivd‐1(tm6784); bcat‐1(hh58)* double mutants compared with *bcat‐1(hh58)* single mutants (Figure 3d) and emerged as a key metabolite for the genetic interaction between *ivd‐1* and *bcat‐1*, in contrast to the extensive metabolic reprogramming of *bcat‐1(hh58)*. Interestingly, carnitine is clinically supplemented to excrete toxic intermediate metabolites that are produced in some BCAA metabolic disorders such as MSUD, IVA, MBDD, or IBDD (Knerr et al., 2012; Villani et al., 2017). It is possible that the loss of function of *bcat‐1* triggers an adaptive metabolic regulation to reduce the amounts of intermediate metabolites produced by impairments of downstream BCAA enzymes.

### 3.4 Increased BCAA intermediates inhibit ubiquitin‐dependent protein degradation

**FIGURE 4:**
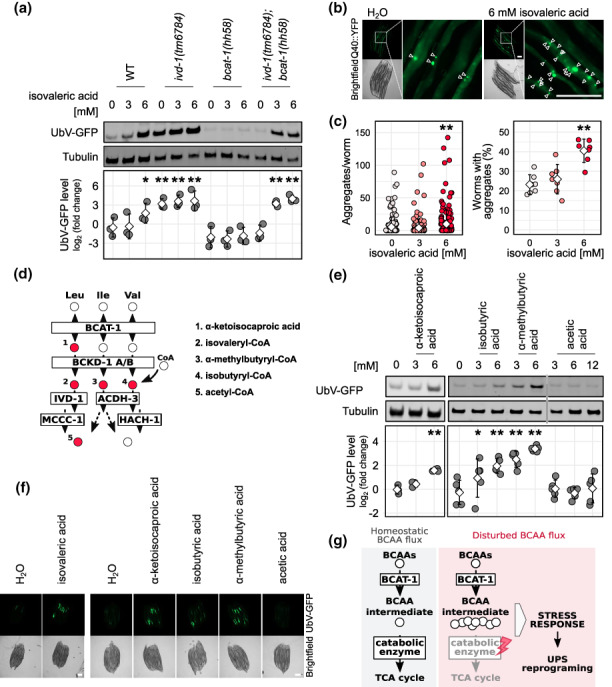
Increased BCAA intermediates inhibit ubiquitin‐dependent protein degradation. (a, e) Western blot analysis of adult worm lysates immunoblotted for GFP (UbV‐GFP) and tubulin as a loading control. Below the blot, quantification of all experimental replicates displayed as the log_2_ of the UbV‐GFP intensity normalized on tubulin and relative to the wild‐type (WT) control. Means as white squares and standard deviations as error bars. A statistically significant difference in relation to control was calculated with pairwise *t*‐tests and indicated with * for *p*‐value ≤0.05, ** for *p*‐value ≤0.01. (a) Wild‐type (WT) and mutant worms were treated with indicated concentrations of isovaleric acid, and H_2_O as control. Three experimental replicates. (b) Fluorescent and brightfield micrographs of immobilized worms expressing Q40::YFP in intestine treated with isovaleric acid, arrowheads indicate protein aggregates. Scale bars: 200 μm. (c) Quantification of protein aggregation in worms expressing Q40::YFP as in (b), with indicated isovaleric acid concentration. The number of aggregates for each worm (top) and the percentage of worms showing at least one aggregate (bottom) are reported. Means as white squares and standard deviations as error bars. Seven experimental replicates with 11 to 30 worms each. Statistically significant difference compared with WT was calculated with Wilcoxon rank sum test with Bonferroni *p*‐value correction, **p‐value ≤0.01. (d) BCAA metabolism schematic view, numbered intermediates (filled with red) are indicated on the right. (e) Growth medium was supplemented with indicated concentrations of carboxylic acids deriving from the metabolites highlighted in (d). Three and five experimental replicates for α‐ketoisocaproic acid and the other metabolites, respectively. (f) Fluorescent and brightfield micrographs of immobilized worms treated with 6 mM of the indicated carboxylic acids as in (a) and (e), scale bar: 200 μm. (g) Impaired BCAA catabolism downstream of the initial transamination step leads to elevated BCAA intermediates (red box), which might trigger an adaptive stress response that includes transcriptional downregulation of UPS components. Consequently, certain classes of proteins might be protected from proteasomal degradation, contributing to a general metabolic reprogramming that influences multiple molecular networks including central metabolism, cell cycle regulation, gene expression, and proteostasis.

Because acetyl‐carnitine was increased in *ivd‐1(tm6784); bcat‐1(hh58)* (Figure 3d) and has been reported as a marker of adequate carnitine supplementation in the clinical treatment of isovaleric acidemia (Itoh et al., 1996), we wondered whether increased isovaleric acid underline lower UPS functionality in *ivd‐1(tm6784)* mutants. Like all BCAA‐derived organic acids, isovaleric acid is difficult to detect by LC–MS because it is highly reactive and has a low *m*/*z* ratio. Therefore, we chose to add isovaleric acid directly to the worm medium. Indeed, the addition of isovaleric acid to the growth medium was sufficient to inhibit the degradation of UbV‐GFP in wild‐type worms. Moreover, isovaleric acid also induced UbV‐GFP accumulation in *ivd‐1(tm6784); bcat‐1(hh58)* mutants, suggesting that the *bcat‐1(hh58)* mutation can suppress *ivd‐1(tm6784)‐*induced UbV‐GFP accumulation by lowering isovaleric acid levels (Figure 4a). The UbV‐GFP level remained unchanged in *bcat‐1(hh58)* mutants supplemented with isovaleric acid, possibly due to higher detoxification by carnitine (Figure 3d). Because IVD‐1 is functional in *bcat‐1(hh58)* mutants, the concentration of isovaleric acid could also be reduced by its binding to CoA and further oxidation to produce acetyl‐CoA, thereby bypassing the initial defective transamination step. Moreover, isovaleric acid supplementation exacerbated the aggregation of the metastable polyglutamine (polyQ) expansion protein Q40::YFP in the intestine of day six adult worms (Figure 4b,c), suggesting that the reduction in proteasomal activity negatively affects the degradation of protein aggregates during aging. Considering that depletion not only of *ivd‐1* but also of other BCAA metabolic enzymes affected the UPS (Figure 1b,h), we investigated whether other intermediate metabolites affect UbV‐GFP substrate turnover (Figure 4d). Strikingly, the addition of three other BCAA‐derived carboxylic acids, α‐ketoisocaproic acid, isobutyric acid, and α‐methylbutyric acid, triggered the accumulation of UbV‐GFP similarly to isovaleric acid (Figure 4e,f). In contrast, acetic acid, even at higher doses, had no effect, confirming that the UPS is specifically regulated by BCAA intermediates and not by general acidification. We hypothesize that disruption of BCAA metabolism causes accumulation of intermediates, which in turn affects proteasome substrate degradation, possibly as part of an adaptive stress response that can be abrogated by inhibition of the initial BCAA transamination (Figure 4g).

## 4 DISCUSSION

BCAA metabolism has been linked to numerous physiological processes, including energy regulation, lipid storage, protein synthesis, and proteolysis (Biswas et al., 2019; Neinast et al., 2019; White et al., 2021). The physiological role of BCAAs and related metabolites has attracted particular interest in the scientific community, especially in light of controversial findings suggesting that both increased and decreased BCAA levels are associated with longevity (D'Antona et al., 2010; Juricic et al., 2020; Mansfeld et al., 2015; Richardson et al., 2021; Solon‐Biet et al., 2019; Trautman et al., 2022; Valerio et al., 2011; White & Newgard, 2019). Here, we demonstrate the important role of BCAA metabolism in the functional reprogramming of proteasomal degradation (Figure 4g). Specifically, we found that alterations in the initial BCAA transamination reaction and downstream catabolic reactions affect the UPS in opposite ways (Figure 1a,b,h). Our data suggest that elevated concentrations of BCAA intermediates are sufficient to affect ubiquitin‐dependent proteolysis (Figure 4a,e,f). Moreover, high concentrations of isovaleric acid promote the aggregation of metastable proteins (Figure 4b,c), which normally begin to aggregate in early adulthood in *C. elegans* (Labbadia & Morimoto, 2015). Because protein aggregation is associated with neurodegenerative diseases and is known to increase with age (Labbadia & Morimoto, 2015; Walther et al., 2015), we speculate that increased BCAA intermediates such as isovaleric acid may accelerate aging. Conversely, silencing of the BCAA pathway by loss‐of‐function of *bcat‐1(hh58)* promotes proteasomal function and potentially reduces the amount of BCAA intermediates (Figure 1e–h, 3d, 4a). The identified role of BCAA intermediates in UPS regulation suggests that dysregulation of the UPS contributes to the pathological course of BCAA metabolic disorders. Considering that the UPS functionality declines with age (Lopez‐Otin et al., 2013; Pohl & Dikic, 2019; Saez & Vilchez, 2014), the reported UPS regulation may contribute to the physiological role of BCAAs in aging.

Since *ivd‐1(tm6784)* mutants have a similar concentration of BCAAs as wild‐type worms (Figure S5d), we hypothesize that a bioactive role of BCAA intermediates influencing proteostasis might partially explain the controversial findings about the different levels of BCAAs in longevity. In contrast to BCAAs, little is known about the biological significance of BCAA derivatives besides energy production. However, clinical data from patients with BCAA hereditary disorders indicate the importance of maintaining physiological levels of BCAA derivatives (Villani et al., 2017). Altered concentrations of BCAA intermediates might not only derive from genetic mutations classified as hereditary BCAA disorders but also natural single nucleotide polymorphisms, highlighting the need for personalized nutrition interventions that consider the genetic background of each individual (Trautman et al., 2022). Although the concentrations we used to supplement these metabolites most likely resemble pathological conditions caused by genetic diseases, it is intriguing to speculate that also minor changes in the levels of these metabolites might influence aging in healthy individuals. Once bound to CoA, the BCAA derivative metabolites are trapped inside mitochondria (Neinast et al., 2019), raising the question of how these metabolites might influence molecular processes taking place in the cytosol such as the UPS. Intriguingly, a recent study identified 3‐hydroxyisobutyrate, a catabolic intermediate of valine that lost CoA, as a paracrine regulator of vascular fatty acid transport that favors insulin resistance in mice (Jang et al., 2016). Not only this finding supports a possible bioactive role of BCAA intermediates but also opens the possibility that CoA could be removed from other BCAA intermediates, leaving them free to exit mitochondria and act as paracrine molecules.

Mechanistically, BCAA intermediates could act as signaling molecules that affect transcription of UPS components, possibly reshaping proteasomal composition and reducing ubiquitin‐dependent degradation in favor of other proteolytic pathways (Figure 2d). Most *ivd‐1(tm6784)‐*downregulated proteasomal subunits belong to the regulatory 19S subunits rather than the 20S core particle (Figure S4d), suggesting alternative proteasome substrate specificity rather than general UPS downregulation. Of the regulated proteasome subunits, RPN‐6.1 has been reported to play a critical role in proteasome proteolytic activity and longevity (Vilchez et al., 2012). Moreover, overexpression of *rpn‐6.1* was able to suppress the proteolytic defects of worms with impaired IVD‐1 (Segref et al., 2014). These results support the possibility that alterations in BCAA metabolism may influence the rate of aging by exploiting the proteasome function. Thus, BCAA intermediates could reduce the degradation of specific protein classes, including CoA‐transferases, and promote the turnover of proteins involved in nutrient storage and lipid transport (Figure 2e,f). We have previously shown that depletion of *ivd‐1* does not affect the total content of ubiquitylated substrates or the enzymatic activity of the proteasome (Segref et al., 2014). In this context, it is interesting to note that BCAA metabolites can trigger protein acylation (Anderson et al., 2017; Wagner et al., 2017), which could target transcription factors and even UPS components such as E3 ubiquitin ligases or proteasomal subunits. Moreover, inhibition of proteasomal activity by BCAA intermediates might be related to the essential role of the 26S proteasome in amino acid recycling (Suraweera et al., 2012). Considering that an *ivd‐1* loss‐of‐function mutation does not affect lifespan and another metabolic mutation affecting the UPS increases longevity (Segref et al., 2014), the observed UPS regulation may result from positive feedback to mild mitochondrial stress, also called mitohormesis (Ristow & Schmeisser, 2014). *bcat‐1(hh58)*, which displays higher proteolysis and increased concentration of BCAAs (Figure 1e,f,h; Figure S5d), is characterized by strong reproductive and developmental defects (Figure S2c–e), supporting the idea that elevated BCAAs are detrimental for health (Conway, 2020; Neinast et al., 2019; Peng et al., 2020; Trautman et al., 2022; White & Newgard, 2019). Nevertheless, the contribution of BCAA intermediates in the regulation of the UPS described here opens future directions of study to unravel the complex regulatory network involving BCAA metabolism.

Alterations in BCAA metabolism are commonly associated with pathological conditions, including not only congenital BCAA metabolic disorders, but also diabetes, cancer, and neurodegeneration, including Alzheimer's disease (Conway, 2020; Neinast et al., 2019; Peng et al., 2020; White & Newgard, 2019). Previous studies in *C. elegans* showed a conserved relationship between *bcat‐1* and aging (Mansfeld et al., 2015). Moreover, defective BCAA transamination in neurons has been shown to exacerbate neurodegeneration and motility defects in a *C. elegans* model of Parkinson's disease (Mor et al., 2020). Although essential functions are retained, the loss‐of‐function *bcat‐1(hh58)* allele appears to lose more protein functions compared with RNAi treatment (Figure 1c–h). Accordingly, the proteomics data showed a very low amount of BCAT‐1 in the mutant worms, comparable to the amounts of the corresponding protein in the *ivd‐1(tm6784)* and *hecd‐1(tm2371)* deletion alleles, respectively (Figure S5e). We hypothesize that the new hypomorphic allele *bcat‐1(hh58)* may help to further elucidate the physiological role of BCAA metabolism in aging and neurodegeneration in a multicellular organism. In this context, it will be important to perform subcellular and tissue‐specific studies to clarify the localization of BCAT‐1 and other BCAA enzymes in nematodes. In mammals, BCAT exists in two specialized forms: the cytosolic BCAT1, which is mainly expressed in neurons, and the ubiquitous mitochondrial BCAT2 (Neinast et al., 2019). It would be interesting to confirm the existence of a second BCAT isoform of *C. elegans* previously predicted by sequence, namely Y44A6D.5, and to evaluate the possible evolutionary link with higher organisms. However, the embryonic lethality resulting from knock‐out of *bcat‐1* (Figure S1d) suggests that Y44A6D.5, if biologically relevant, would not compensate for the loss of BCAT‐1. Considering that proteostasis decline often correlates with the progression of pathology and aging (Hipp et al., 2019; Hoppe & Cohen, 2020; Labbadia & Morimoto, 2015), the impact of BCAA intermediates on UPS activity and proteostasis forms the basis for developing therapies to treat pathological conditions associated with impaired BCAA metabolism, identifying the proteasome and BCAT as two possible therapeutic targets in combination with BCAA dietary interventions to promote healthy aging.

## AUTHOR CONTRIBUTIONS

Sonia Ravanelli designed, performed, and analyzed the experiments. Andrea Annibal carried out the metabolomics and, together with Sonia Ravanelli and Adam Antebi, interpreted the data. Qiaochu Li conducted protein aggregation studies. Thorsten Hoppe supervised the experimental design and the data interpretation. Aleksandra Trifunovic contributed to data analysis and provided resources. Sonia Ravanelli and Thorsten Hoppe wrote the manuscript. All authors discussed the results and commented on the manuscript.

## CONFLICT OF INTEREST

The authors declare no competing interests.

## Supporting information


Figure S1
Figure S2
Figure S3
Figure S4
Figure S5
Figure S6

Table S5

Appendix S1

## Data Availability

RNA‐seq data have been deposited in NCBI's Gene Expression Omnibus with dataset identifier GSE185451. The mass spectrometry proteomics data have been deposited to the ProteomeXchange Consortium via the PRIDE repository with the dataset identifier PXD028286. The metabolomics data are reported in Table S5. Further information and requests for resources and reagents should be directed to and will be fullfilled by the lead contact, Thorsten Hoppe (thorsten.hoppe@uni-koeln.de).
