# Supplementary material for: Reprograming of proteasomal degradation by branched chain amino acid metabolism
Source: Aging Cell. 2022 Sep 27;21(12):e13725. doi: 10.1111/acel.13725 (PMC9741504; doi:10.1111/acel.13725)
Supplement: Supplementary file 1 — Figure S1 Figure S2 Figure S3 Figure S4 Figure S5 Figure S6 [file ACEL-21-e13725-s003.pdf]

Figure S1

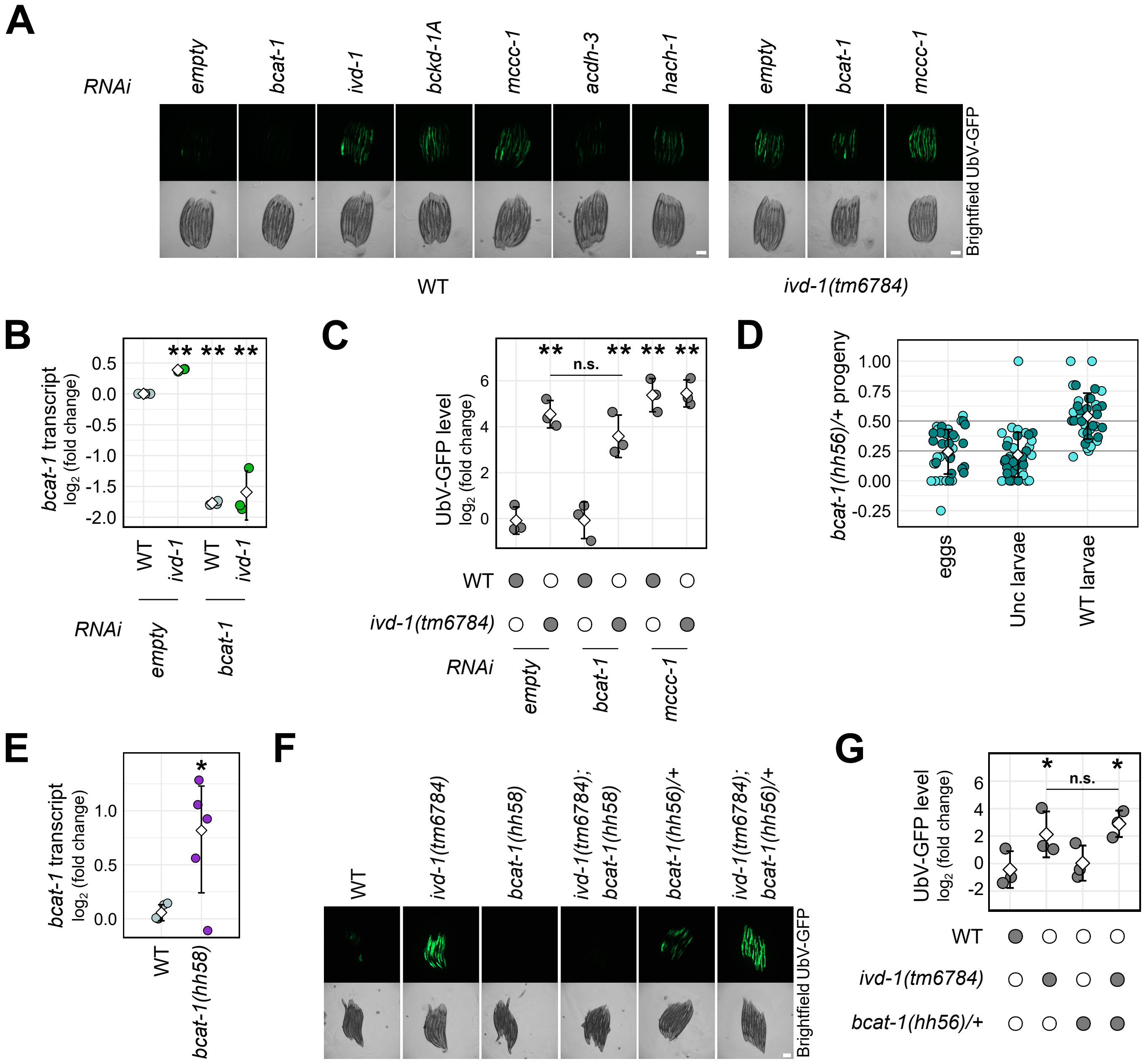

A
RNAi
empty
bcat-1
ivd-1
bckd-1A
mccc-1
acdh-3
hach-1
empty
bcat-1
mccc-1
UbV-GFP
Brightfield
WT
ivd-1(tm6784)
B
bcat-1 transcript
log2 (fold change)
** ** **
WT
ivd-1
WT
ivd-1
RNAi
empty
bcat-1
C
UbV-GFP level
log2 (fold change)
** n.s. ** ** **
WT
ivd-1(tm6784)
RNAi
empty
bcat-1
mccc-1
D
bcat-1(hh56)/+ progeny
eggs
Unc larvae
WT larvae
E
bcat-1 transcript
log2 (fold change)
*
WT
bcat-1(hh58)
F
WT
ivd-1(tm6784)
bcat-1(hh58)
ivd-1(tm6784); bcat-1(hh58)
bcat-1(hh56)/+
ivd-1(tm6784); bcat-1(hh56)/+
UbV-GFP
Brightfield
G
UbV-GFP level
log2 (fold change)
* n.s. *
WT
ivd-1(tm6784)
bcat-1(hh56)/+

Figure S2

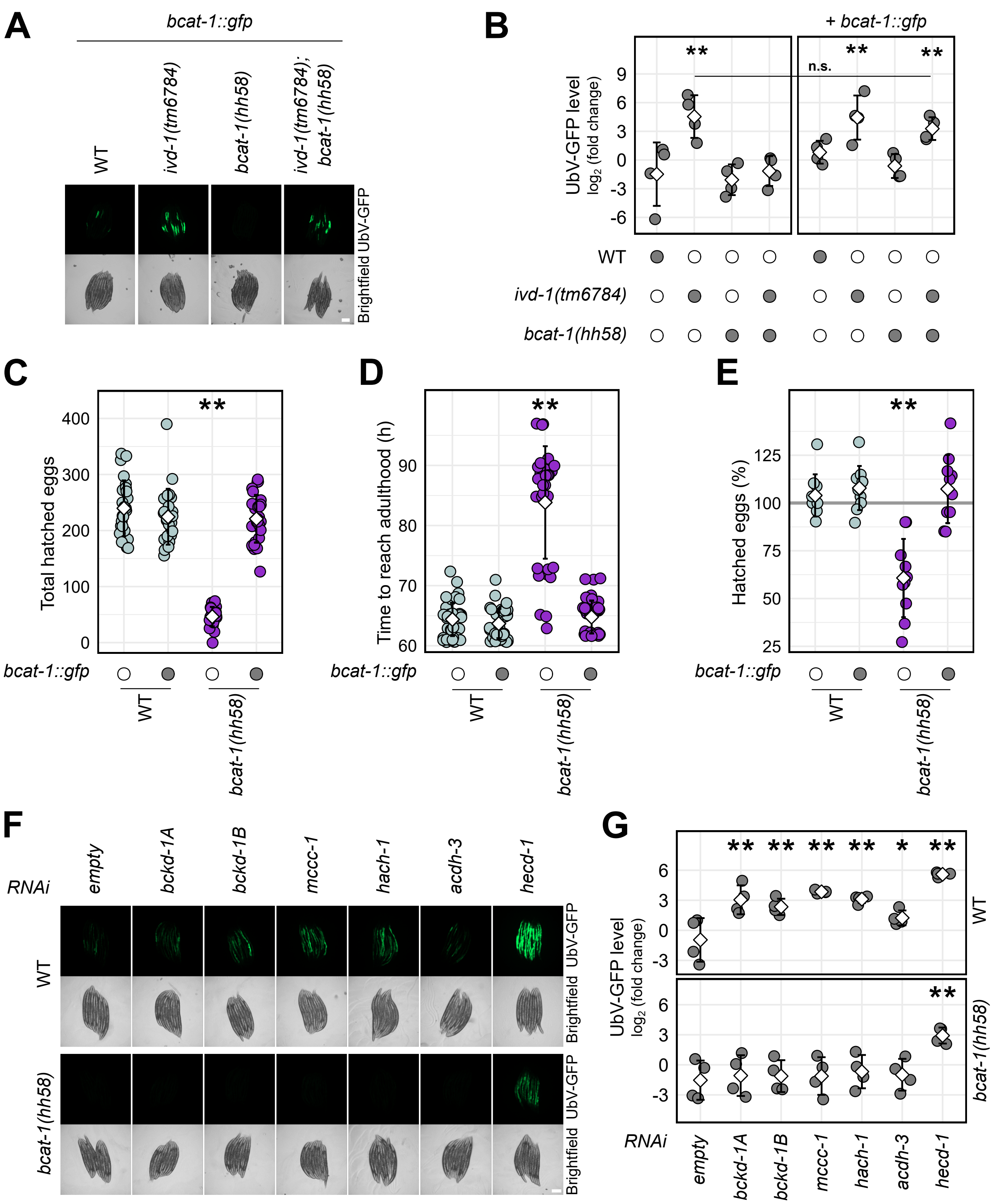
A
bcat-1::gfp
WT
ivd-1(tm6784)
bcat-1(hh58)
ivd-1(tm6784); bcat-1(hh58)
UbV-GFP
Brightfield
B
+ bcat-1::gfp
UbV-GFP level
log2 (fold change)
n.s.
**
WT
ivd-1(tm6784)
bcat-1(hh58)
C
Total hatched eggs
bcat-1::gfp
WT
bcat-1(hh58)
D
Time to reach adulthood (h)
E
Hatched eggs (%)
F
RNAi
empty
bckd-1A
bckd-1B
mccc-1
hach-1
acdh-3
hecd-1
G
*

Figure S3

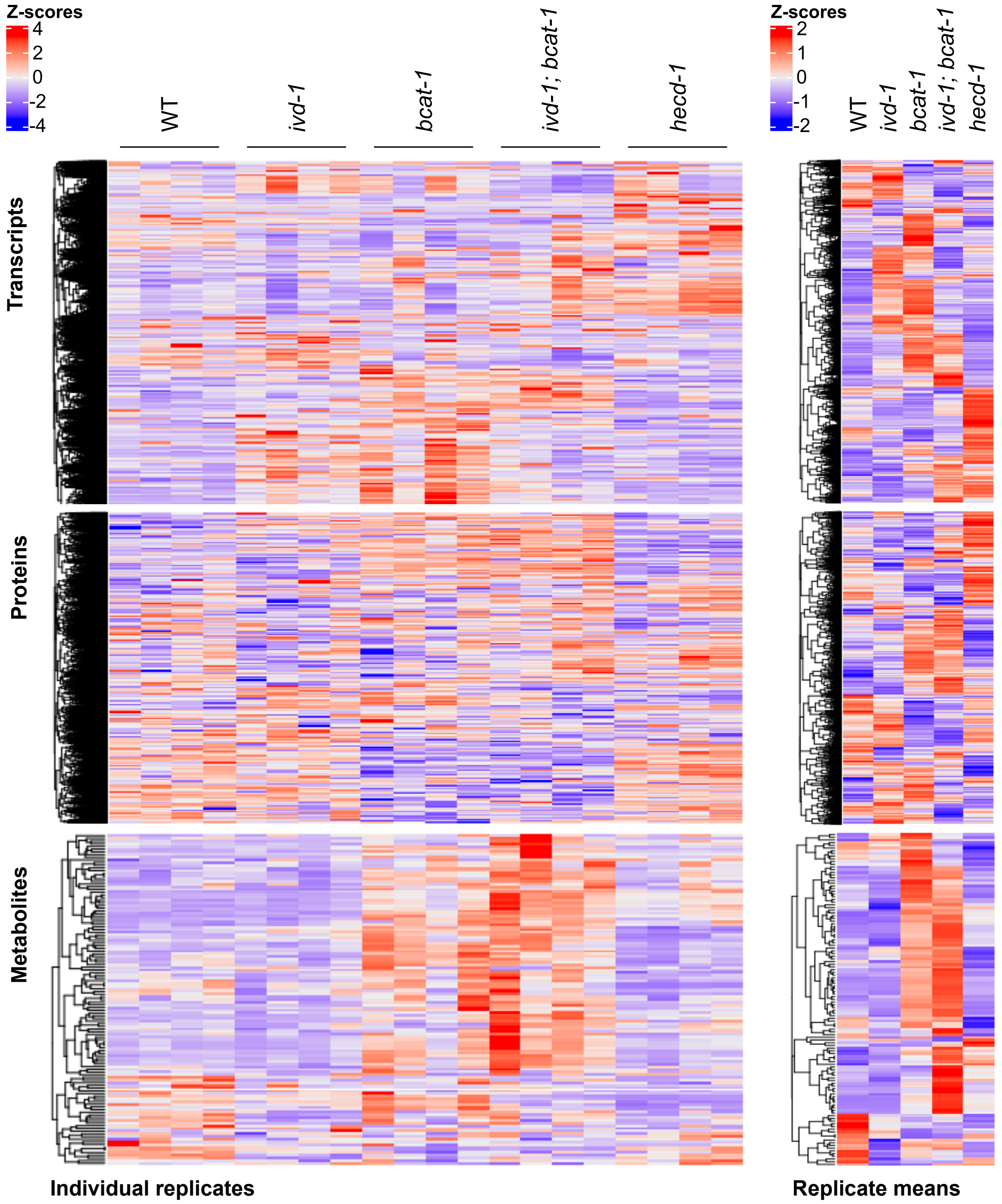

Z-scores
4
2
0
-2
-4
WT
ivd-1
bcat-1
ivd-1; bcat-1
hecd-1
Z-scores
2
1
0
-1
-2
WT
ivd-1
bcat-1
ivd-1; bcat-1
hecd-1
Transcripts
Proteins
Metabolites
Individual replicates
Replicate means

Figure S4

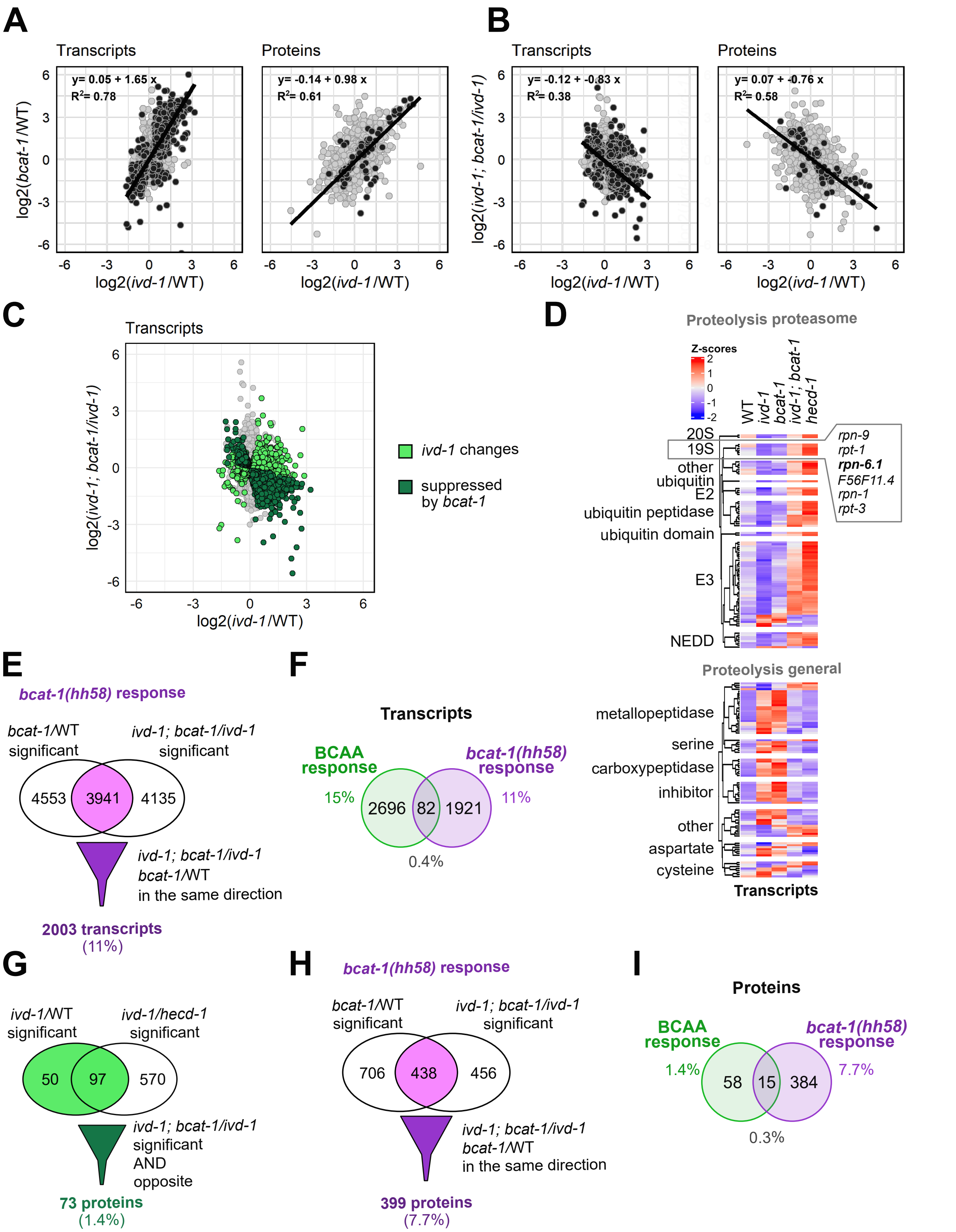

A
Transcripts
Proteins
y= 0.05 + 1.65 x
R²= 0.78
y= -0.14 + 0.98 x
R²= 0.61
log2(bcat-1/WT)
log2(ivd-1/WT)
B
Transcripts
Proteins
y= -0.12 + -0.83 x
R²= 0.38
y= 0.07 + -0.76 x
R²= 0.58
log2(ivd-1; bcat-1/ivd-1)
log2(ivd-1/WT)
C
Transcripts
log2(ivd-1; bcat-1/ivd-1)
log2(ivd-1/WT)
ivd-1 changes
suppressed by bcat-1
D
Proteolysis proteasome
Z-scores
WT
ivd-1
bcat-1
ivd-1; bcat-1
hecd-1
20S
19S
other ubiquitin
E2
ubiquitin peptidase
ubiquitin domain
E3
NEDD
rpn-9
rpt-1
rpn-6.1
F56F11.4
rpn-1
rpt-3
Proteolysis general
metallopeptidase
serine
carboxypeptidase
inhibitor
other
aspartate
cysteine
Transcripts
E
bcat-1(hh58) response
bcat-1/WT significant
ivd-1; bcat-1/ivd-1 significant
4553
3941
4135
ivd-1; bcat-1/ivd-1
bcat-1/WT
in the same direction
2003 transcripts
(11%)
F
Transcripts
BCAA response
bcat-1(hh58) response
15%
11%
2696
82
1921
0.4%
G
ivd-1/WT significant
ivd-1/hecd-1 significant
50
97
570
ivd-1; bcat-1/ivd-1
significant
AND
opposite
73 proteins
(1.4%)
H
bcat-1(hh58) response
bcat-1/WT significant
ivd-1; bcat-1/ivd-1 significant
706
438
456
ivd-1; bcat-1/ivd-1
bcat-1/WT
in the same direction
399 proteins
(7.7%)
I
Proteins
BCAA response
bcat-1(hh58) response
1.4%
7.7%
58
15
384
0.3%

Figure S5

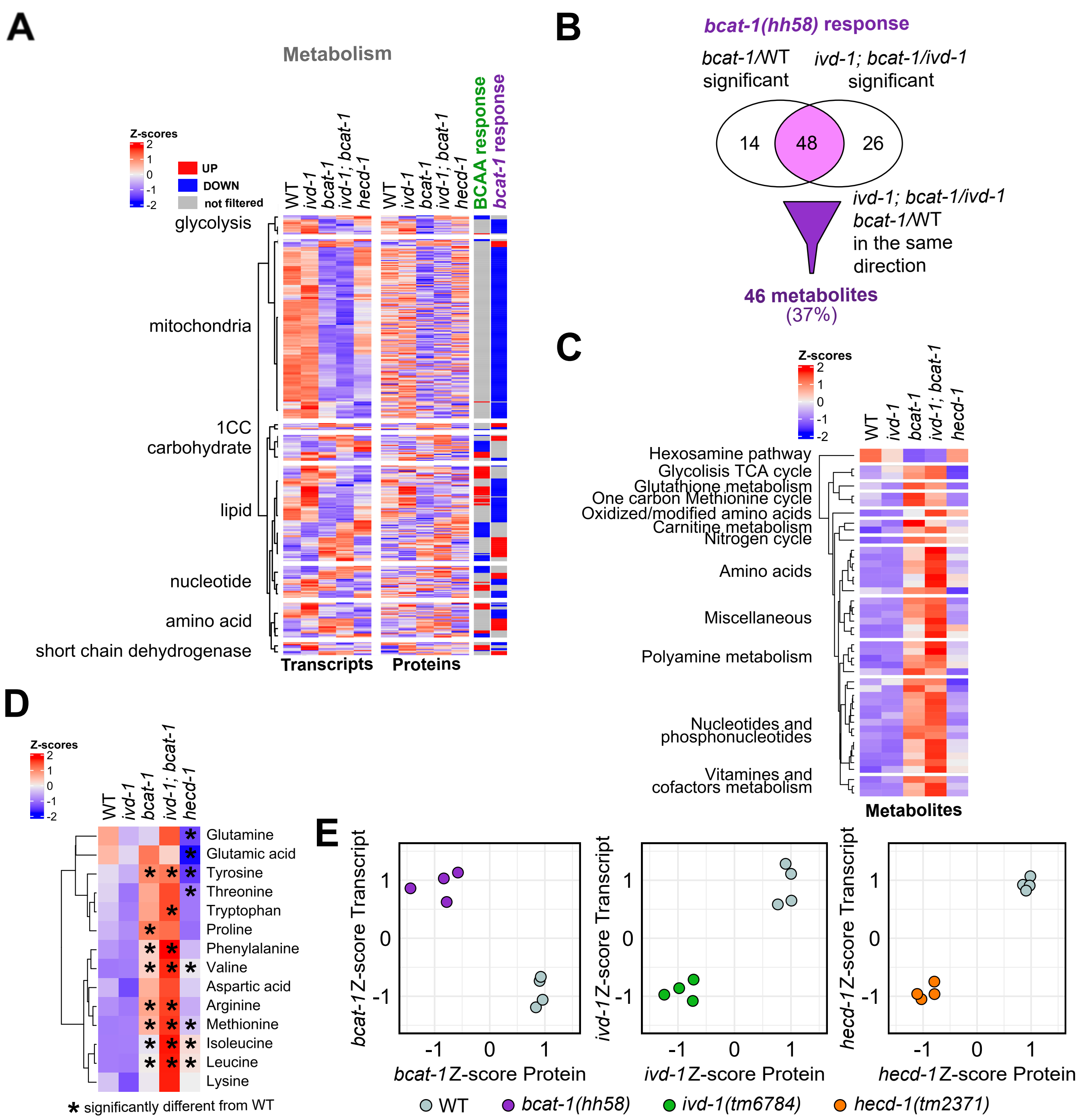

A
Metabolism
Z-scores
UP
DOWN
not filtered
WT
ivd-1
bcat-1
ivd-1; bcat-1
hecd-1
BCAA response
bcat-1 response
glycolysis
mitochondria
1CC
carbohydrate
lipid
nucleotide
amino acid
short chain dehydrogenase
Transcripts
Proteins
B
bcat-1(hh58) response
bcat-1/WT significant
ivd-1; bcat-1/ivd-1 significant
14
48
26
ivd-1; bcat-1/ivd-1
bcat-1/WT
in the same direction
46 metabolites
(37%)
C
Hexosamine pathway
Glycolisis TCA cycle
Glutathione metabolism
One carbon Methionine cycle
Oxidized/modified amino acids
Carnitine metabolism
Nitrogen cycle
Amino acids
Miscellaneous
Polyamine metabolism
Nucleotides and phosphonucleotides
Vitamines and cofactors metabolism
Metabolites
D
Glutamine
Glutamic acid
Tyrosine
Threonine
Tryptophan
Proline
Phenylalanine
Valine
Aspartic acid
Arginine
Methionine
Isoleucine
Leucine
Lysine
* significantly different from WT
E
bcat-1 Z-score Transcript
bcat-1 Z-score Protein
ivd-1 Z-score Transcript
ivd-1 Z-score Protein
hecd-1 Z-score Transcript
hecd-1 Z-score Protein
WT
bcat-1(hh58)
ivd-1(tm6784)
hecd-1(tm2371)

Figure S6

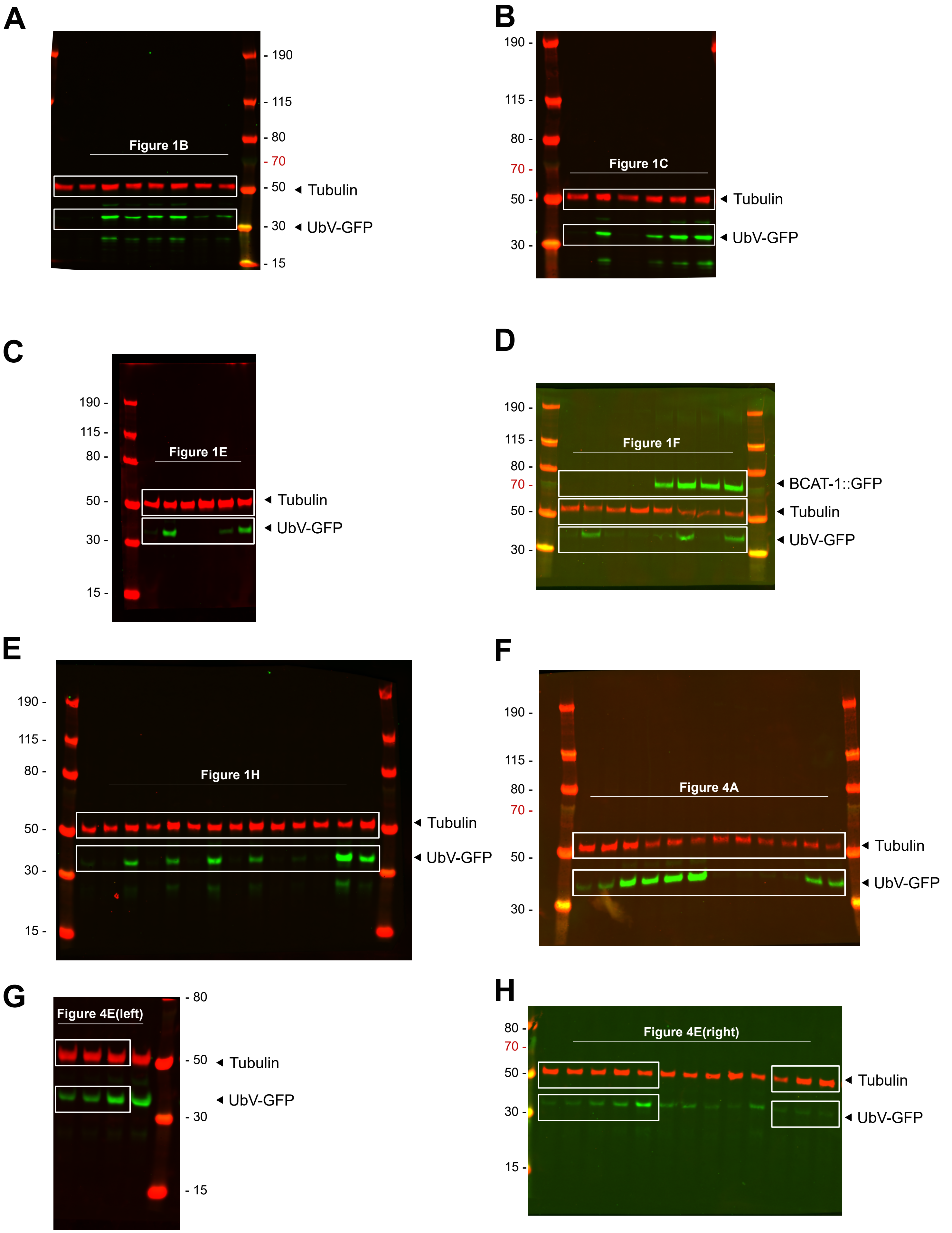
A
Figure 1B
190
115
80
70
50
Tubulin
30
UbV-GFP
15
B
Figure 1C
190
115
80
70
50
Tubulin
30
UbV-GFP
C
Figure 1E
190
115
80
50
Tubulin
UbV-GFP
30
15
D
Figure 1F
190
115
80
70
BCAT-1::GFP
50
Tubulin
UbV-GFP
30
E
Figure 1H
190
115
80
50
Tubulin
UbV-GFP
30
15
F
Figure 4A
190
115
80
70
Tubulin
50
UbV-GFP
30
G
Figure 4E(left)
80
50
Tubulin
UbV-GFP
30
15
H
Figure 4E(right)
80
70
50
Tubulin
30
UbV-GFP
15

Supplementary Figure Legends

Figure S1. *bcat-1* Is Essential and Its Inhibition Does Not Affect the UPS

(A, F) Fluorescent and brightfield micrographs of immobilized worms, scale bars: 200 µm. (A) Same strains and conditions as in Figures 1B and 1C. (B) Transcript level of *bcat-1* relative to wild-type (WT) treated with empty vector control measured by qRT-PCR. Means of 3 technical replicates are reported for 3 independent experimental replicates. Same strains and conditions as in Figure 1C. (B, E) Fold changes calculated as $2^{-\Delta\Delta Cq}$. Means as white squares and standard deviations as error bars. Statistically significant difference calculated with Tukey's Honest Significant Difference (HSD) combined with one-way ANOVA and indicated in relation to control with * for P-value ≤ 0.05, ** for P-value ≤ 0.01. (C, G) Western blot quantifications displayed as the $\log_2$ of the intensity normalized on tubulin and relative to the wild-type (WT) control. Filled circles denote genetic mutations. Means as white squares and standard deviations as error bars. Statistically significant difference in relation to control, or as highlighted, were calculated with pairwise t-tests and indicated with * for P-value ≤ 0.05, ** for P-value ≤ 0.01, “n.s” for P-value > 0.05. (C) Quantification relative to Figure 1C. (D) Quantification of *bcat-1(hh56)* lethality, as the proportion of unhatched eggs (*bcat-1(hh56)/bcat-1(hh56)*), viable larvae with uncoordinated (Unc) phenotype (*tmC24/tmC24*), and phenotypically wild-type (WT) larvae (*bcat-1(hh56)/tmC24*). Light and dark blue identify two independent lines isolated from crossing *bcat-1(hh56)/+* with balancer strain FX30253. 19 and 20 biological replicates for each line. (E) Transcript level of *bcat-1* quantified with qRT-PCR relative to WT. Means of at least 3 technical replicates are reported for 5 independent experimental replicates. (F) Same strains and conditions as in Figure 1E. (G) Quantification relative to Figure 1E. *bcat-1(hh58)* quantification is reported in Figure S2B.

Figure S2. *bcat-1(hh58)* Loss-of-Function Mutation Suppresses the Proteolytic Defects of Downstream BCAA Impairments

(A, F) Fluorescent and brightfield micrographs of immobilized worms, scale bars: 200 µm. (A) Strains expressing *bcat-1::gfp* reported in Figure 1F. (B, G) Western blot quantifications displayed as the $\log_2$ of the intensity normalized on tubulin and relative to the wild-type (WT) control. Means as white squares and standard deviations as error bars. Statistically significant difference in relation to control, or as highlighted, were calculated with pairwise t-tests and indicated with * for P-value ≤ 0.05, ** for P-value ≤ 0.01, “n.s” for P-value > 0.05. (B) Quantification relative to Figure 1F. Filled circles denote genetic mutations, strains expressing *bcat-1::gfp* are reported in the right quadrant. (C) Total number of viable progeny quantified for individual worms. 2 experimental replicates pooled together to have minimum 24 worms for wild-type (WT) and *bcat-1(hh58)*, respectively. (D) Generation time of individual worms, considered as the time to reach adulthood from egg. 2 experimental replicates pooled together to have minimum 34 worms for each strain. (E) Viability quantification as proportion of viable progeny on the total number of eggs laid by 1 adult worm in 4 hours, 10 worms for each strain. (C-E) Means as white square and standard deviations as error bars are displayed for each strain. Filled circles denote *bcat-1::gfp* transgene expression. Statistically significant difference compared to WT were calculated with Tukey's Honest Significant Difference (HSD) combined with one-way ANOVA, indicated as ** for p ≤ 0.01. (F) Same strains and conditions as in Figure 1H. (G) Quantification relative to Figure 1H. The top quadrant reports wild-type (WT) worms, the bottom *bcat-1(hh58)* mutants.

Figure S3. Transcriptome, Proteome and Metabolome Modulation by BCAA Metabolism

Heatmaps of Z-scores from the complete omics datasets (transcriptomics, proteomics, metabolomics) as in Figure 2A. 4 individual replicates for each strain (left) and relative mean (right). The reported mutant alleles are *ivd-1(tm6784), bcat-1(hh58)*, and *hecd-1(tm2371).*

Figure S4. The Restored UPS Activity in *ivd-1(tm6784)*; *bcat-1(hh58)* Is Mediated by an Adaptive Response Rather than Broad *bcat-1(hh58)*-Dependent Regulation

(A, B) Scatterplots of fold changes relative to transcripts (left) and proteins (right), with darker fill indicating significantly different fold changes for both axes (P-value ≤ 0.05). Linear regression with relative formula and R^2 are indicated in black. The reported alleles are wild-type (WT), *ivd-1(tm6784)* and *bcat-1(hh58).* (C) Scatterplot showing fold changes relative to Figure 2C. (D) Heatmap with Z-scores relative to transcript levels of genes in the Wormcat categories “Proteolysis Proteasome” and “Proteolysis general” that were identified by the filtering reported in Figure 2C. The reported mutant alleles are *ivd-1(tm6784), bcat-1(hh58)*, and *hecd-1(tm2371).* Gene names of 19S proteasome subunits are indicated. (E) Venn diagram of transcripts that are significantly regulated by *bcat-1(hh58)* in wild-type (WT) and *ivd-1(tm6784)* background (pink) that follow the same direction (violet) (P-value ≤ 0.05). (F) Intersection between regulated transcripts identified in Figure 2C (BCAA response) and S4E (*bcat-1(hh58)* response). (G) Venn diagram of *ivd-1(tm6784)*-significantly regulated proteins compared with wild-type (WT) in light green and *hecd-1(tm2371)*, which are suppressed by *bcat-1(hh58)* (dark green) (P-value ≤ 0.05). (H) Proteins regulated by *bcat-1(hh58)* filtered as in S4E. (I) Intersection between regulated proteins identified in S4G (BCAA response) and S4H (*bcat-1(hh58)* response).

Figure S5. BCAA Transamination Induces Broad Metabolic Reprogramming

(A) Heatmaps with Z-scores relative to transcript and protein levels of genes in the Wormcat gene category “Metabolism” that were transcriptionally regulated either according to Figure 2C (BCAA response) or Figure S4E (*bcat-1* response), indicated on the right as up- (red) or down-regulated (blue) or not filtered (grey). Clustering according to transcript levels and split according to Wormcat category 2. (A, C) The reported mutant alleles are *ivd-1(tm6784), bcat-1(hh58)*, and *hecd-1(tm2371).* (B) Venn diagram of metabolites that are significantly regulated by *bcat-1(hh58)* in wild-type (WT) and *ivd-1(tm6784)* background (pink) that follow the same direction (violet) (P-value ≤ 0.05). (C) Heatmap with Z-scores relative to the level of the 46 metabolites filtered in B. (D) Heatmap with Z-scores relative to the level of each amino acid, * denotes significant changes compared to wild-type (WT). (E) Analysis of protein and transcript level of respective mutated genes reported as scatterplots of protein (x) and transcript (y) Z-scores calculated on each mutant strain compared with wild-type (WT).

Figure S6: Uncropped Western Blots

(A-H) Un-cropped Western Blots, with boxes highlighting the portions reported in the indicated figures. Ladder with respective kDa sizes are reported on the side. Primary antibody incubation was done mixing the rabbit antibody against tubulin diluted 1:5000, and the mouse antibody against GFP diluted 1:7000. Detection was carried out after incubation with mixture of Li-Cor secondary antibodies 680RD donkey anti-rabbit (displayed in red) and 800CW donkey anti-mouse (displayed in green), both diluted 1:15000. Antibody details in Table S2.
