# Supplementary material for: Reprograming of proteasomal degradation by branched chain amino acid metabolism
Source: Aging Cell. 2022 Sep 27;21(12):e13725. doi: 10.1111/acel.13725 (PMC9741504; doi:10.1111/acel.13725)
Supplement: Supplementary file 3 — Appendix S1 [file ACEL-21-e13725-s002.pdf]

Supplementary Experimental Procedures

RNA Extraction and qRT-PCR

Synchronized worms by egg-prep were harvested at day 1 of adulthood after washing with M9 at least twice to remove bacteria. Worm pellets were flash-frozen in liquid nitrogen and stored at -80°C. To extract total RNA, 1 ml TRIzol (Ambion) was added to the worm pellets together with 200 µl zirconia beads and homogenization was conducted with Precellys 24-Dual cell homogenizer (Peqlab). After addition of 1-bromo-3-chloropropane, mixing and phase separation by centrifugation, aqueous supernatant was mixed with the same volume of 70% ethanol and total RNA was isolated with Quiagen RNeasy Mini kit following the manufacturer's instructions. Using High-Capacity cDNA Reverse Transcription Kit (Applied Biosystems), 1500 ng of isolated RNA was retro-transcribed to cDNA. Gene expression levels were measured via quantitative real time PCR (qRT-PCR) with Bio-Rad CFX96 Real-Time PCR Detection System and the Luna Universal qPCR Master Mix (New England Biolabs), following the manufacturer instructions. Quantification cycles (Cq) were obtained by the Bio-Rad CFX Manager 3.1 software and subsequently analyzed with Rstudio to calculate the fold changes: $2^{-\Delta\Delta Cq}$ as described previously (Livak & Schmittgen, 2001). Actin served as internal control, primer efficiencies were checked by creation of a standard curve and retro-transcribed products lacking retrotranscriptase in the reaction were included to confirm absence of DNA in the originally isolated RNA. Primer sequences are reported in Table S1.

CRISPR-Cas9 Mutation

The guide RNAs (crRNA) were selected combining outputs of the two online tools CHOPCHOP (version 2) and Spacer Scoring for CRISPR (SSC) (Labun et al., 2016; Xu et al., 2015) and guidelines reported by Paix et al., 2017. Universal structural RNA (tracrRNA), *dpy-10* and *bcat-1* crRNAs, single-stranded DNA oligonucleotide (ssODN) template for the homologous recombination, and Cas9 nuclease (New England Biolabs) were supplemented with HEPES and KCl. The obtained injection mix was centrifuged at full speed in a tabletop centrifuge and supernatant was injected with the aid of a FemtoJet (Eppendorf) in the gonad

of several young adult worms immobilized with Holocarbon oil on a dried 2% agarose pad. After multiple microinjections, worms were rehydrated with a drop of M9 and transferred to OP50-seeded growth plates to recover. After 4/5 days, adult worms displaying either roller or dumpy phenotypes were isolated and let laying eggs for another day before genotyping to identify *bcat-1* mutant alleles. After PCR amplification of the interested *bcat-1* coding region, NheI restriction digestion (New England BioLabs) served to identify integration of the premature stop codon. Selected isolates were confirmed by sequencing and propagated avoiding dumpy and roller worms to exclude *dpy-10* mutations. In case of *bcat-1(hh56)*, heterozygous worms were isolated and subsequently crossed with FX30253 balancer strain (Dejima et al., 2018) to maintain heterozygous *bcat-1(hh56)/+* and outcrossed 4 times. *bcat-1(hh58)* was outcrossed 6 times.

Viable Progeny Quantification

To quantify the total viable progeny, 10-20 L4 larvae were singled for each strain and transferred every day. The total number of viable progenies was obtained summing up the number of viable worms present in each plate after the parental worm was transferred. Data from 2 independent experiments were pooled to have a minimum of 24 replicates after censoring parental worms that died before laying the entire progeny.

Generation Time Quantification

The generation time or time to reach adulthood was quantified as the time needed from egg to reach adulthood. Adult worms were let laying eggs for 4 hours; after 2 days, 20 to 25 larvae were isolated from the progeny and monitored regularly to note the time when the first progeny eggs were laid. 2 experimental replicates were pooled together to have minimum 34 technical replicates for each strain, after censoring worms that died before reaching adulthood.

Embryonic Lethality Quantification

To assess the lethality penetrance of the *bcat-1(hh56)* mutation, heterozygous *bcat-1(hh56)/tmC24* worms isolated from two independent lines were analyzed. 20 L4 larvae were singled from each line and removed from plates after 2 hours, unhatched eggs were immediately counted, while viable progeny was quantified

after 3 days, distinguishing for phenotypically wild-type (WT) from uncoordinated (Unc), which indicate two copies of the balancer *tmC24*. Dead embryos were obtained by subtracting the total number of viable progeny from the total number of laid eggs. All phenotypically wild-type larvae were confirmed to be heterozygous for *bcat-1(hh56)* by genotyping. Worms that were not alive when the progeny was quantified were censored. Proportions of unhatched eggs, phenotypically wild-type and Unc larvae are reported in the graph. The same procedure was applied for *bcat-1(hh58)* mutants except that the single adult worms were left 4 hours on plates to lay eggs. For each strain 10 adult worms were singled, the total number of eggs counted after having removed the adults and viable larvae were counted 3 days after. Worms that were not alive when the progeny was quantified were censored.

Sample Processing for Transcriptomics

Trizol was supplemented to worm pellets to extract RNA as described for qRT-PCR (final dilution 50-200 ng/µl). Libraries were prepared using the Illumina® Stranded TruSeq® RNA sample preparation Kit. Library preparation started with 2 µg total RNA. After poly-A selection (using poly-T oligo-attached magnetic beads), mRNA was purified and fragmented using divalent cations under elevated temperature. The RNA fragments underwent reverse transcription using random primers. This was followed by second strand cDNA synthesis with DNA Polymerase I and RNase H. After end repair and A-tailing, indexing adapters were ligated. The products were then purified and amplified (14 PCR cycles) to create the final cDNA libraries. After library validation and quantification (Agilent 2100 Bioanalyzer), equimolar amounts of library were pooled. The pool was quantified by using the Peqlab KAPA Library Quantification Kit and the Applied Biosystems 7900HT Sequence Detection System. The pool was sequenced with Illumina NovaSeq6000 sequencing instrument with a PE100 protocol. RNAseq raw data were analysed using a QuickNGS pipeline (Wagle et al., 2015). This workflow system provided a basic read quality check using FastQC (version 0.10.1) and read statistics using SAMtools (version 0.1.19). The basic data processing of the QuickNGS pipeline consists of a splicing-aware alignment using Tophat2 (version 2.0.10) followed by reference-guided transcriptome reassembly with Cufflinks2 (version 2.1.1). The QuickNGS pipeline calculated read count means, fold change and P-values with DEseq2 (version 1.4.5) and gene expression for the individual samples with Cufflinks2 (version 2.1.1) as fragments per kilobase of transcript per million (FPKMs), in both cases using genomic annotation

from the Ensembl database, version 93. All data pre-processing was done with R version 3.1.1 and Bioconductor version 3.0.

Sample Processing for Proteomics

Worm pellets were supplemented with urea buffer (8 M in 50 mM TEAB + 1x Roche protease inhibitor cocktail), sonicated, centrifuged for 15 min at 20000 x g and protein content was quantified with Pierce BCA protein assay (ThermoFisher Scientific). Samples were diluted to have 50 µg of proteins; DTT, and chloracetamide were added to a final concentration of 5 mM and 40 mM, respectively. Peptide digestion was conducted first with lysyl endopeptidase (Lys-C) and, after diluting the samples to a final urea concentration of 1.2 M with trypsin protease. All samples were analyzed on a Q Exactive Plus Orbitrap (ThermoScientific) mass spectrometer that was coupled to an EASY nLC (ThermoScientific). Peptides were loaded with solvent A (0.1% formic acid in water) onto an in-house packed analytical column (50 cm — 75 µm I.D., filled with 2.7 µm Poroshell EC120 C18, Agilent). Peptides were chromatographically separated at a constant flow rate of 250 nl/min and the following gradient: 3-5% solvent B (0.1% formic acid in 80 % acetonitrile) within 1.0 min, 5-30% solvent B within 91.0 min, 30-50% solvent B within 17.0 min, 50-95% solvent B within 1.0 min, followed by washing and column equilibration. Data-dependent acquisition (DDA) library runs were acquired from distinct pools of the sample groups. The MS1 survey scan was acquired from 300-1750 m/z at a resolution of 70,000. The top 10 most abundant peptides were isolated within a 2.0 Th window and subjected to HCD fragmentation with normalized collision energy of 27%. The AGC target was set to 5e5 charges, allowing a maximum injection time of 55 ms. Product ions were detected in the Orbitrap at a resolution of 17,500. Precursors were dynamically excluded for 20.0 s. For the acquisition of the samples the mass spectrometer was operated in data-independent mode. For MS1 and MSMS scans the maximum IT was restricted to 60 ms and the AGC target was set to 1e6 charges. The MS1 scan was acquired from 400-1220 m/z at a resolution of 35,000. MSMS scans were acquired in data-independent acquisition (DIA) mode using 25 x 24 m/z windows covering the mass range from m/z 400 to m/z 1000 at a resolution of 17,500. The default charge state for the MS2 was set to 3. Stepped normalized collision energy was set to 27%. The MSMS spectra were acquired in centroid mode. A spectrum library was generated by searching a UniProt UP000001940 *C. elegans* fasta file (26754 entries) with PulsarX in Spectronaut 13

(Bruderer et al., 2015). DDA runs of high pH reversed phase HPLC fractionated protein digests and in gel digests of SDS page separated protein lysates were used for library generation. Protein quantification analysis was performed in Perseus (version 1.6.7.0) (Tyanova et al., 2016), including $\log_2$ transformation, filtering for valid values occurring in 4 out of 4 replicates in at least one strain, imputation of missing values from normal distribution and two-sample tests.

Sample Processing for Untargeted Metabolomics

Worm pellets were homogenized using a Qiagen tissue lyser for 30 min at 4 °C. Protein concentration was determined using a BCA kit. A volume of worm lysate, which corresponds to 150 µg of proteins for each sample was subjected to Bligh and Dyer extraction (chloroform: methanol, 2:1) for 1 h at 4 °C as previously reported on our work (Annibal et al., 2019). Samples were centrifuged at maximum speed for 5 min at 4 °C and supernatant was transferred into a new tube for drying. Before LC injections samples were reconstituted in 10% aqueous acetonitrile. Analytes were separated using an UHPLC system (Vanquish, Thermo Fisher Scientific) coupled to an HRAM mass spectrometer (Q-Exactive Plus, Thermo Fischer Scientific) as previously reported (Annibal et al., 2021). Briefly, 2 µl of the sample extracts were injected into a X Select HSS T3 XP column, 100 Å, 2.5 µm, 2.1 mm x 100 mm (Waters), using a binary system A water with 0.1% formic acid, B: acetonitrile with 0.1 formic acid with a flowrate of 0.1 ml/min and the column temperature was kept at 30 °C. Gradient elution was conducted as follow: isocratic step at 0.1 % eluent B for 0.3 min, gradient increase up to 2% eluent B in 2 min, then increase up to 30% eluent B in 6 min and to 95% eluent B in 7 min, isocratic step at 95% eluent B for 2 min. Gradient decreases to 0.1 % eluent B in 3 min and held at 0.1% eluent B for 5 min. Mass spectra were recorded from 100-800 *m/z* at a mass resolution of 70,000 at *m/z* 400 in both positive and negative ion mode using data-dependent acquisition (Top 3, dynamic exclusion list 10 seconds). Tandem mass spectra were acquired by performing CID (isolation 1,5 u, stepped collision energy 20 and 80 NCE). MS data analysis was performed using Xcalibur software 4.0. Quantification was performed using Trace finder 4.1, using genesis detection algorithm, nearest RT, S/N threshold 8, min peak height (S/N) equal to 3, peak S/N cutoff 2.00, valley rise 2%, valley S/N 1.10. Metabolite search was performed using Compound discoverer 2.0 and m/z Cloud as online database, considering precursor ions with a deviation > 5 ppm, 0.3 min maximum retention time shift, minimum peak

intensity 100000, intensity tolerance 10, FT fragment mass tolerance 0.0025 Da, group covariance [%] less than 30, P-value less than 0.05 and area Max greater or equal to 10000. Metabolites are correctly identified when at least two specific fragments are found in the MS2 spectra. Because of the high mass accuracy >3 ppm, predicted elemental compositions of the unknown features were submitted to online databases such as Chemspider (http://www.chemspider.com/), HMDB (http://www.hmdb.org/), KEGG (http://www.genome.jp/kegg/), METLIN (http://metlin.scripps.edu/). Raw data were also analyzed using XCMS online (https://xcmsonline.scripps.edu).

Omics data analysis

The pre-processed transcriptomics, proteomics and metabolomics datasets were analyzed with R (version 4.0.0), in Rstudio environment (version 1.2.5033). Filtering, Z-score calculation and scatterplots were conducted with the aid of tidyverse packages. Heatmaps were generated using ComplexHeatmap package (Gu et al., 2016). To compare expression levels of transcripts and proteins of the respective gene, the two datasets were merged using the Wormbase IDs. Except for the metabolomics data, P-values refer to adjusted P-values. The metabolic map reported in Figure 3A was extracted from multiple maps generated by Pathview (Luo et al., 2017), using a simplified dataset obtained assigning 1 for significantly up-regulated, -1 for significantly downregulated and 0 for not significant changes of transcripts and metabolites relative to $\log_2$(*ivd-1(tm6784)*/WT) and $\log_2$(*ivd-1(tm6784);bcat-1(hh58)/ivd-1(tm6784)*). Gene enrichment was performed with Wormcat (genome v1) (Holdorf et al., 2020) in case of regulated transcripts and with GOrilla (Eden et al., 2009) in case of regulated proteins, where the entire set of detected proteins was submitted as background list of genes and molecular function was selected as ontology. The GOrilla enrichment score is defined as (b/n)/(B/N), where N is the total number of genes in the background list, B is the total number of genes associated with a specific GO term, n is a flexible cutoff, and b the number of identified genes associated with a specific GO term (Eden et al., 2009).

Table S1. Genotyping and qRT-PCR oligo sequences

Allele/Transgene	Forward	Reverse
**Genotyping**		
sur-5p::UbV-GFP	TTTTGGTACCATGCAAATCTTCGT CAAAACGTTG	CGTTAGTTAGTAGAACTCAG
bcat-1 CRISPR-Cas9 (+NheI HF digestion, New England BioLabs)	AATGTACTGCCTCCATCGGC	GGGTCCAATGGCTTGGTCTT
ivd-1 (tm6784)	TCCATTCGCACAGATACACA	GTATGCTGACAAGATTGACAAG
	CCATTCAATACATACTTGTCTCC	
bcat-1p::bcat-1::GFP	TTAATGACATTCAGTACGGC	CGTTAGTTAGTAGAACTCAG
**qRT-PCR**		
bcat-1	ATCAAGGCTTATGGCCTCCG	ACTGTCTCCAATCTGGCTGC
act-1	CCACCATGTACCCAGGAATT	AGAGGGAAGCGAGGATAGAT

Table S2. Antibodies

Antibody	Dilution	Source	Identifier
Living Colors A.v. GFP Monoclonal Antibody [JL-8] raised in mouse	1:7000	Clontech	Cat. # 632380
Rabbit monoclonal alpha Tubulin antibody [EP1332Y]	1:5000	Abcam	Cat. # ab52866; RRID: AB_869989
IRDye® 680RD Donkey anti-Rabbit IgG Secondary Antibody	1:15000	Li-Cor	Cat. # 926-68073; RRID: AB_10954442
IRDye® 800CW Donkey anti-Mouse IgG Secondary Antibody	1:15000	Li-Cor	Cat. # 926-32212; RRID: AB_621847

Table S3. Chemical Supplements

Compound	Source	Identifier
isovaleric acid, 3-methylbutanoic acid	Sigma-Aldrich	Cat.# 129542
sodium α-ketoisocaproic acid, sodium 4-methyl-2-oxovaleric acid	Sigma-Aldrich	Cat. # K0629
isobutyric acid, 2-methylpropionic acid	Sigma-Aldrich	Cat. # 58360
α-methylbutyric acid, 2-methylbutyric acid	Sigma-Aldrich	Cat. # 193070
acetic acid glacial	VWR Chemicals	Cat. # 20104.334

Table S4. CRISPR-Cas9 oligonucleotides

Oligonucleotide	Sequence	Purchased by
universal tracrRNA	AACAGCAUAGCAAGUUAAAAUAAGGCUAGUCCGUUAUCAA CUUGAAAAAGUGGCACCGAGUCGGUGCUUUUUUU	Dharmacon
bcat-1(hh56) crRNA	ugucuccaaucuggcugcgg	Dharmacon
bcat-1(hh56) ssODN	cttcaaccttgtcggatcaaggcttatggcctccgGCTAGCCcagccagattggaga cagtaccaagagaggaaatt	Metabion (HPLC purified)
bcat-1(hh58) crRNA	ugcguggagcaacucgcgac	Dharmacon
bcat-1(hh58) ssODN	ccagaaatacacacaataatgccagcaatcctgtcCAGCTAGCgcgagttgctcc acgcaccttcaaccttgtcggat	Metabion (HPLC purified)
dpy-10 crRNA	GCUACCAUAGGCACCACGAG	Dharmacon
dpy-10 ssODN	CACTTGAACTTCAATACGGCAAGATGAGAATGACTGGAAAC CGTACCGCATGCGGTGCCTATGGTAGCGGAGCTTCACATG GCTTCAGACCAACAGCCTAT	Metabion (HPLC purified)

References

Annibal, A., Tam, H., Latza, C., & Antebi, A. (2019). Comparison of ESI-MS/MS and APCI-MS methods for the quantification of folic acid analogs in C. elegans. *Journal of Mass Spectrometry*, *54*(4), 316–327. https://doi.org/10.1002/jms.4337

Annibal, A., Tharyan, R. G., Schonewolff, M. F., Tam, H., Latza, C., Auler, M. M. K., & Antebi, A. (2021). Regulation of the one carbon folate cycle as a shared metabolic signature of longevity. *Nature Communications*, *12*(1), 3486. https://doi.org/10.1038/s41467-021-23856-9

Bruderer, R., Bernhardt, O. M., Gandhi, T., Miladinović, S. M., Cheng, L.-Y., Messner, S., Ehrenberger, T., Zanotelli, V., Butscheid, Y., Escher, C., Vitek, O., Rinner, O., & Reiter, L. (2015). Extending the Limits of Quantitative Proteome Profiling with Data-Independent Acquisition and Application to Acetaminophen-Treated Three-Dimensional Liver Microtissues*[S]. *Molecular & Cellular Proteomics*, *14*(5), 1400–1410. https://doi.org/10.1074/mcp.M114.044305

Dejima, K., Hori, S., Iwata, S., Suehiro, Y., Yoshina, S., Motohashi, T., & Mitani, S. (2018). An Aneuploidy-Free and Structurally Defined Balancer Chromosome Toolkit for Caenorhabditis elegans. *Cell Reports*, *22*(1), 232–241. https://doi.org/10.1016/j.celrep.2017.12.024

Eden, E., Navon, R., Steinfeld, I., Lipson, D., & Yakhini, Z. (2009). GOrilla: A tool for discovery and visualization of enriched GO terms in ranked gene lists. *BMC Bioinformatics*, *10*(1), 48. https://doi.org/10.1186/1471-2105-10-48

Gu, Z., Eils, R., & Schlesner, M. (2016). Complex heatmaps reveal patterns and correlations in multidimensional genomic data. *Bioinformatics*, *32*(18), 2847–2849. https://doi.org/10.1093/bioinformatics/btw313

Holdorf, A. D., Higgins, D. P., Hart, A. C., Boag, P. R., Pazour, G. J., Walhout, A. J. M., & Walker, A. K. (2020). WormCat: An Online Tool for Annotation and Visualization of Caenorhabditis elegans Genome-Scale Data. *Genetics*, *214*(2), 279–294. https://doi.org/10.1534/genetics.119.302919

Labun, K., Montague, T. G., Gagnon, J. A., Thyme, S. B., & Valen, E. (2016). CHOPCHOP v2: A web tool for the next generation of CRISPR genome engineering. *Nucleic Acids Research*, *44*(W1), W272–W276. https://doi.org/10.1093/nar/gkw398

Livak, K. J., & Schmittgen, T. D. (2001). Analysis of relative gene expression data using real-time quantitative PCR and the 2(-Delta Delta C(T)) Method. *Methods (San Diego, Calif.)*, *25*(4), 402–408. https://doi.org/10.1006/meth.2001.1262

Luo, W., Pant, G., Bhavnasi, Y. K., Blanchard, S. G., Jr, & Brouwer, C. (2017). Pathview Web: User friendly pathway visualization and data integration. *Nucleic Acids Research*, *45*(W1), W501–W508. https://doi.org/10.1093/nar/gkx372

Paix, A., Folkmann, A., & Seydoux, G. (2017). Precision genome editing using CRISPR-Cas9 and linear repair templates in C. elegans. *Methods*, *121–122*, 86–93. https://doi.org/10.1016/j.ymeth.2017.03.023

Tyanova, S., Temu, T., Sinitcyn, P., Carlson, A., Hein, M. Y., Geiger, T., Mann, M., & Cox, J. (2016). The Perseus computational platform for comprehensive analysis of (prote)omics data. *Nature Methods*, *13*(9), 731–740. https://doi.org/10.1038/nmeth.3901

Wagle, P., Nikolić, M., & Frommolt, P. (2015). QuickNGS elevates Next-Generation Sequencing data analysis to a new level of automation. *BMC Genomics*, *16*(1), 487. https://doi.org/10.1186/s12864-015-1695-x

Xu, H., Xiao, T., Chen, C.-H., Li, W., Meyer, C. A., Wu, Q., Wu, D., Cong, L., Zhang, F., Liu, J. S., Brown, M., & Liu, X. S. (2015). Sequence determinants of improved CRISPR sgRNA design. *Genome Research*, *25*(8), 1147–1157. https://doi.org/10.1101/gr.191452.115
